# Obesity-associated inflammation triggers an autophagy–lysosomal response in adipocytes and causes degradation of perilipin 1

**DOI:** 10.1038/s41419-019-1393-8

**Published:** 2019-02-11

**Authors:** Liping Ju, Junfeng Han, Xiaoyan Zhang, Yujie Deng, Han Yan, Congrong Wang, Xiaohua Li, Shuqin Chen, Miriayi Alimujiang, Xu Li, Qichen Fang, Ying Yang, Weiping Jia

**Affiliations:** 1 Shanghai Key Laboratory of Diabetes, Shanghai Institute for Diabetes, Shanghai Clinical Medical Centre of Diabetes, Shanghai Key Clinical Centre of Metabolic Diseases, Department of Endocrinology and Metabolism, Shanghai JiaoTong University Affiliated Sixth People’s Hospital, Shanghai, 200233 China; 2 Department of Endocrine and Metabolic Diseases, Institute of Endocrine and Metabolic Diseases, Ruijin Hospital, Shanghai Jiaotong University School of Medicine, Shanghai, 200025 China; 3 Department of Endocrinology and Metabolism, Yangpu Hospital, Tongji University School of Medicine, Shanghai, 200090 China; 4 Department of Endocrinology, The Affiliated Hospital of Qingdao University, Qingdao, 266003 China; 5 Department of Endocrinology, Seventh People’s Hospital of Shanghai University of TCM, Shanghai, 200137 China

## Abstract

In obesity, adipocytes exhibit high metabolic activity accompanied by an increase in lipid mobilization. Recent findings indicate that autophagy plays an important role in metabolic homeostasis. However, the role of this process in adipocytes remains controversial. Therefore, we performed an overall analysis of the expression profiles of 322 lysosomal/autophagic genes in the omental adipose tissue of lean and obese individuals, and found that among 35 significantly differentially expressed genes, 34 genes were upregulated. A large number of lysosomal/autophagic genes also were upregulated in murine 3T3-L1 adipocytes challenged with tumor necrosis factor α (TNFα) (within 24 h), which is in accordance with increased autophagy flux in adipocytes. SQSTM1/p62, a selective autophagy receptor that recognizes and binds specifically to ubiquitinated proteins, is transcriptionally upregulated upon TNFα stimulation as well. Perilipin 1 (PLIN1), a crucial lipid droplet protein, can be ubiquitinated and interacts with SQSTM1 directly. Thus, TNFα-induced autophagy is a more selective process that signals through SQSTM1 and can selectively degrade PLIN1. Our study indicates that local proinflammatory cytokines in obese adipose tissue impair triglyceride storage via autophagy induction.

## Introduction

Macroautophagy (hereafter referred to as autophagy) is a lysosomal degradation pathway that involves the rearrangement of subcellular membranes to sequester cargo for delivery to the lysosome via the fusion of autophagosomes, whereupon the sequestered material is degraded and recycled^1^. Autophagy can be nonselective or selective. Selective autophagy is mediated by autophagic adapter proteins, such as SQSTM1/p62, NBR1, NDP52, and NIX. SQSTM1 is a polyubiquitin chain binding protein that can recognize and bind specifically to ubiquitinated proteins to act as a shuttle protein to selectively sequester ubiquitinated substrates into lysosomes^2^. On the other hand, SQSTM1 itself is degraded by autophagy, and increased levels of the SQSTM1 protein may suggest that autophagic flux is impaired. Thus, SQSTM1 can accumulate either by increasing SQSTM1 transcription or by blocking autophagic flux^3^. SQSTM1-mediated autophagy is involved in diverse cellular processes and may have a clinical impact on several age-related pathologies and inflammatory diseases^4–6^.

Recently, there has been a growing interest in the role of autophagy in adipocyte biology, and studies suggest that autophagy is functionally linked to lipid storage in vitro^7–9^. Autophagy has also been shown to be altered in adipose tissues in obese individuals. However, whether the related autophagy activity is elevated or impaired is debatable^10–13^. Therefore, defining the regulatory mechanism of autophagic activity at the adipocyte level may help us to better understand the events occurring in vivo.

The adipose tissue microenvironment in obesity enters into a proinflammatory state, which can cause adipocyte dysfunction through the actions of cytokines, such as tumor necrosis factor α (TNFα). The overproduction of TNFα within the adipose tissue of obese individuals chronically stimulates lipolysis and impairs triglyceride storage^14^. Obese individuals have a deficiency of perilipin 1 (PLIN1), a lipid droplet-associated protein that promotes lipid droplet formation and inhibits adipocyte lipolysis, even if their adipocytes are larger, and hence obese individuals show an increased basal rate of lipolysis^15^. On the other hand, other studies have established that proinflammatory cytokines can induce autophagy. In human atherosclerotic vascular smooth cells, TNFα plays an important role in the pro-autophagic effect via the c-jun N-terminal kinase^16^. In a *Drosophila melanogaster* malignant tumor model, early-stage tumor growth and invasion are genetically dependent upon tumor necrosis factor and interleukin-6 mediated autophagy within the local tumor microenvironment^17^. However, in obese adipose tissue, whether local proinflammatory cytokines may contribute to adipocyte dysfunction via autophagy remains unclear.

Our current study found that a large number of lysosomal/autophagic genes were transcriptionally upregulated in the omental adipose tissue from obese individuals, which resulted in an increased autophagy activity in adipocytes. The proinflammatory cytokines secreted by macrophages account for this process. Increased autophagy induced by TNFα in adipocytes results in selective degradation of PLIN1 through SQSTM1. Thus, our study shows that proinflammatory cytokines in local adipose tissue can stimulate adipocyte autophagy, which can result in elevated levels of lipolysis, thus impairing triglyceride storage in obese adipose tissues.

## Results

### Lysosomal/autophagic genes were upregulated in the omental adipose tissue from obese individuals

**Fig. 1:**
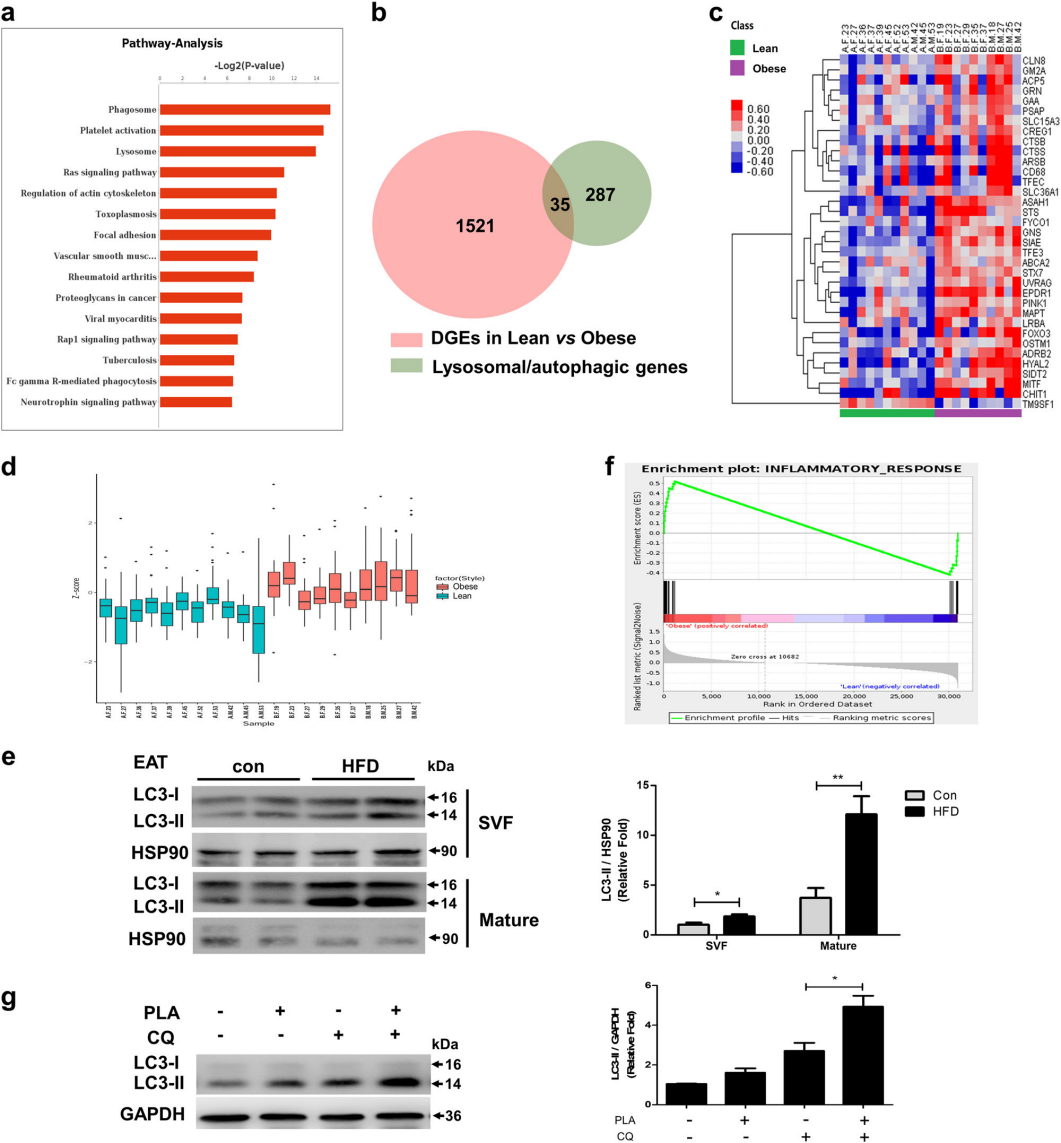
Lysosomal/autophagic genes were upregulated and associated with inflammation **a** Omental adipose tissues were extracted from 11 lean controls and 10 obese individuals to perform RNA-sequencing analysis. Differentially expressed genes (DEGs) were used to perform pathway analysis based on the KEGG database using Fisher’s exact test. **b** Venn diagram showing the overlap between DEGs and 322 mouse genes mainly involved in the lysosomal and/or autophagic pathway. **c** Heatmap analysis displaying the lysosomal/autophagic genes in human omental adipose tissue. **d** Box plots showing human lysosomal/autophagic gene expression based on *z*-score analysis. **e** LC3 content were determined by western blot in the stromal vascular (SVF) and mature adipocytes of epididymal adipose tissue (EAT) from mice fed a high-fat diet (HFD) or standard chow diet for 24 weeks. *n* = 2 for each group, and three mice were pulled together for each sample. **f** RNA-sequencing data were analyzed using Gene set enrichment analysis (GSEA), using the inflammatory response as the gene expression phenotype. This figure shows the enrichment plot for inflammatory response. **g** 3T3-L1 adipocytes were incubated in macrophage-conditioned medium without stimulation (Control-CM) or stimulated with 0.5 mm palmitate for 16 h (PLA-CM) before Chloroquine (CQ) treatment (50 µmol/L). The levels of LC3 were evaluated by immunoblotting cell lysates. *p < 0.05, **p < 0.01, ***p < 0.001

To investigate the alteration of autophagy in adipose tissue under obese conditions, we performed RNA sequence analysis of omental adipose tissue from 11 lean and 10 obese individuals. The clinical characteristics of our study subjects are shown in Supplementary Table 1. To characterize the functional consequences of gene expression changes caused by obesity, differentially expressed genes (DEGs) were identified using the following criteria:^18^ Fold Change >1.2 or <0.833 and a FDR <0.2. As a result,1556 DEGs were identified. Of these DEGs, 874 were upregulated and 682 were downregulated (Supplementary Data File 1). Pathway analysis showed that many of these upregulated genes are members of the phagosome and lysosome pathway (Fig. 1a), suggesting that lysosome/autophagic genes play a role(s) in the progression of obesity. Therefore, the expression patterns of the previously reported 322 lysosomal/autophagic genes^19^ were examined, which have complete homology to their human counterparts. Ultimately, 35 significantly differentially expressed genes were identified (Fig. 1b), including 34 upregulated and 1 downregulated gene (Fig. 1c), whose functions and relationships with lysosome and/or autophagy are shown in Supplementary Table 2. The distribution frequencies are shown in the box plot, and we determined that the z-score of lysosomal/autophagic gene expression in obese individuals was greater than in lean controls (Fig. 1d). These results indicated that a large number of lysosomal/autophagic genes are transcriptionally upregulated in the omental adipose tissue of obese subjects.

### Autophagy flux is induced by proinflammatory macrophage-conditioned medium (CM) in adipocytes

Our previous study found that the protein level of lipidated LC3-II was dramatically higher in both subcutaneous and epididymal fat from mice fed a high-fat diet (HFD) vs. mice fed a standard chow diet^20^. Here, we further determined that the increased LC3-II content was mainly owing to its expression in the mature adipocytes of the epididymal fat (Fig. 1e, Supplementary Figure. 1). In addition, it is well known that obesity is usually accompanied by a state of chronic, low-grade inflammation^21^, and autophagy has been reported to be induced by numerous proinflammatory cytokines^22,23^. In our current study, gene set enrichment analysis revealed a striking enrichment of transcripts involved in the inflammatory response among the genes that were more highly expressed in obesity (Fig. 1f). We next questioned whether obesity-induced local inflammation was involved in the regulation of autophagy in adipocytes. 3T3-L1 adipocytes were exposed to macrophage-conditioned medium, and the autophagic flux was determined. As shown in Fig. 1g, LC3-II accumulated in the presence of chloroquine (CQ, a lysosomal inhibitor), especially in the palmitate stimulated macrophage conditioned medium (PLA-CM) group, which reflected increased autophagic flux under inflammatory conditions. These findings suggested that obesity-related proinflammatory factors promoted autophagy flux in adipocytes.

### Proinflammatory TNFα upregulated lysosomal/autophagic genes within 24 h

**Fig. 2:**
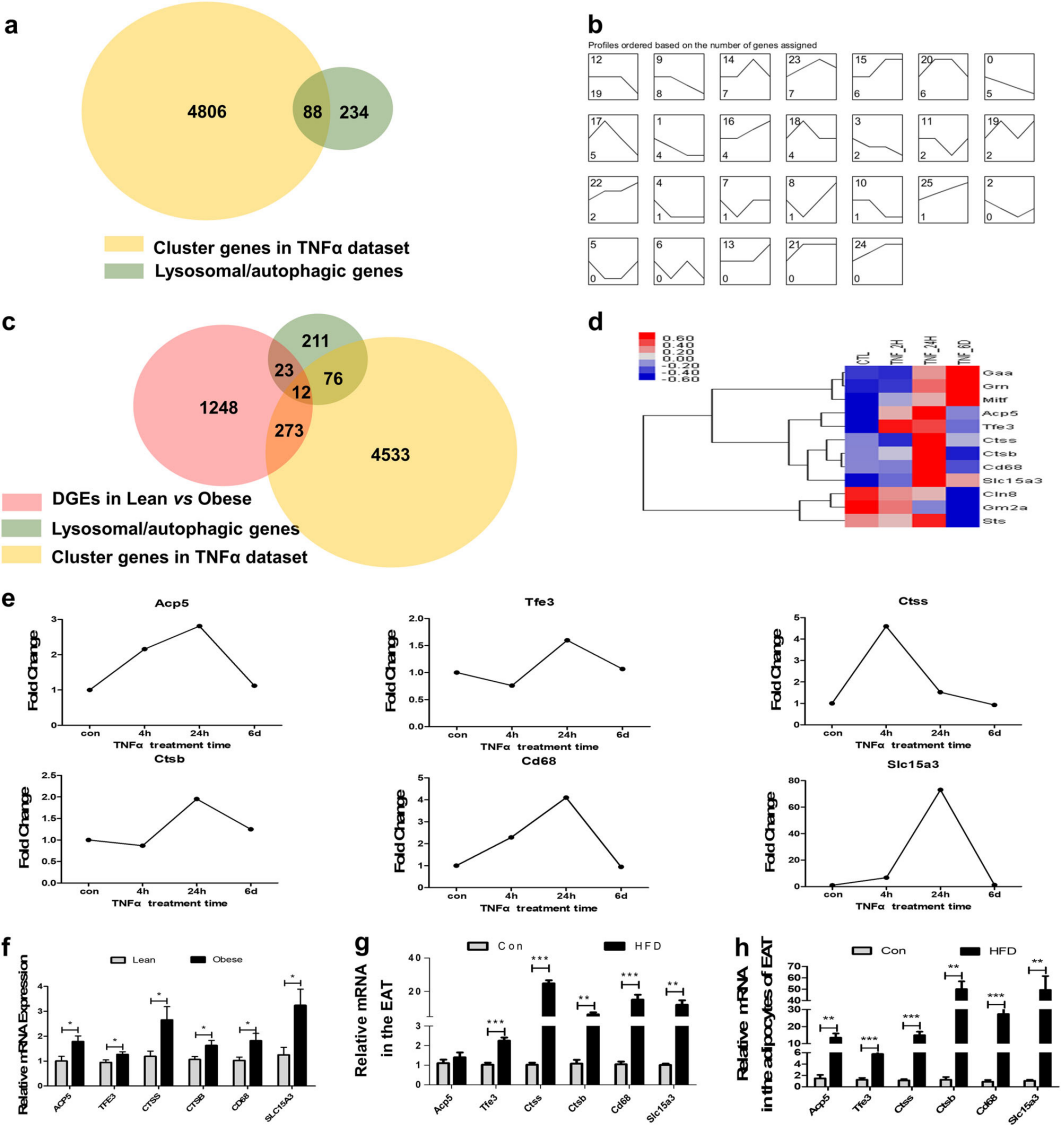
TNFα caused a major upregulation of lysosomal/autophagic genes at 2 and 24 h, some of which is in accordance with the changes in obese subjects **a** Venn diagram showing the overlap between cluster genes in the TNFα data set (GSE62635) and lysosomal/autophagic genes. **b** Cluster analysis of lysosomal/autophagic genes in the TNFα data set. **c** Venn diagram showing the overlap between altered lysosomal/autophagic genes in the human data set and TNFα data set. **d** The heatmap analysis displaying the variation of these genes after TNFα treatment for different times. A red–blue color scale was used to reflect standardized gene expression, with red representing high expression and blue representing low expression (scale shown in the upper left). **e** Results of microarray analysis were further verified in 3T3-L1 adipocytes by qRT-PCR. **f** Lysosomal/autophagic genes were verified by qRT-PCR in human omental adipose tissue. **g–h** The EAT, SVF and mature adipocytes of the EAT were separated from C57BL/6 mice fed HFD or standard chow for 24 weeks. Lysosomal/autophagic genes in the EAT **g** and the mature adipocytes **h** were determined by qRT-PCR (*n* = 6–10). *p < 0.05, **p < 0.01, ***p < 0.001

To comprehensively assess lysosomal/autophagic gene expression changes caused by inflammation, we analyzed the GSE62635 data set^24^, in which 3T3-L1 adipocytes were treated with TNFα for a predetermined period of time (2 h, 24 h, or 6 d). In series cluster analysis, 26 possible profiles were identified, which represent the overall expression patterns (Supplementary Figure. 2). Using Venn diagram analysis, 88 lysosomal/autophagic genes were identified as differentially regulated with TNFα treatment in 3T3-L1 adipocytes (Fig. 2a), which were further assessed by series cluster analysis. As shown in Fig. 2b, there were 29 lysosomal/autophagic genes upregulated at 2 or 24 h, which were subsequently downregulated at 6 d. There were 27 lysosomal/autophagic genes that remained unchanged at 2 or 24 h, but were then downregulated at 6 d. These results suggested that TNFα may facilitate autophagy via upregulation of lysosomal/autophagic gene transcription at early and intermediate stages, whereas long-term stimulation with TNFα may damage the function of the lysosome/autophagy.

To identify common lysosomal/autophagic genes that were important in the pathogenesis of obesity-related inflammation in adipocytes, we constructed a Venn diagram showing lysosomal/autophagic genes identified as DEGs in human omental adipose tissue and 3T3-L1 adipocytes (Fig. 2c); 12 common genes were identified. In 3T3-L1 adipocytes, Acp5, Tfe3, Ctss, Ctsb, Cd68, and Slc15a3 were among these lysosomal/autophagic genes, which were enriched in profile 14, 20, and 23 and were upregulated at early (2 h) and intermediate (24 h) time points, then downregulated at later time points (6 d) (Fig. 2d). They were re-evaluated using qRT-PCR analysis, and the expression of these genes was consistent with the microarray data (Fig. 2e). The expression of these six genes was further investigated by qRT-PCR in human omental adipose tissue and was significantly upregulated in fat tissues from obese individuals (Fig. 2f). Also, after being feed a HFD for 24 weeks, Acp5, Tfe3, Ctss, Ctsb, Cd68, and Slc15a3 mRNA expression was upregulated in adipose tissue from obese mice, particularly in the epididymal fat (Fig. 2g, Supplementary Figure. 3A). The increased expression of these genes was mainly observed in the mature adipocytes vs. the stromal vascular fraction (SVF) (Fig. 2h, Supplementary Figure. 3B).

### Cathepsin B contributed to the pathogenesis of obesity-related inflammation in adipocytes

**Fig. 3:**
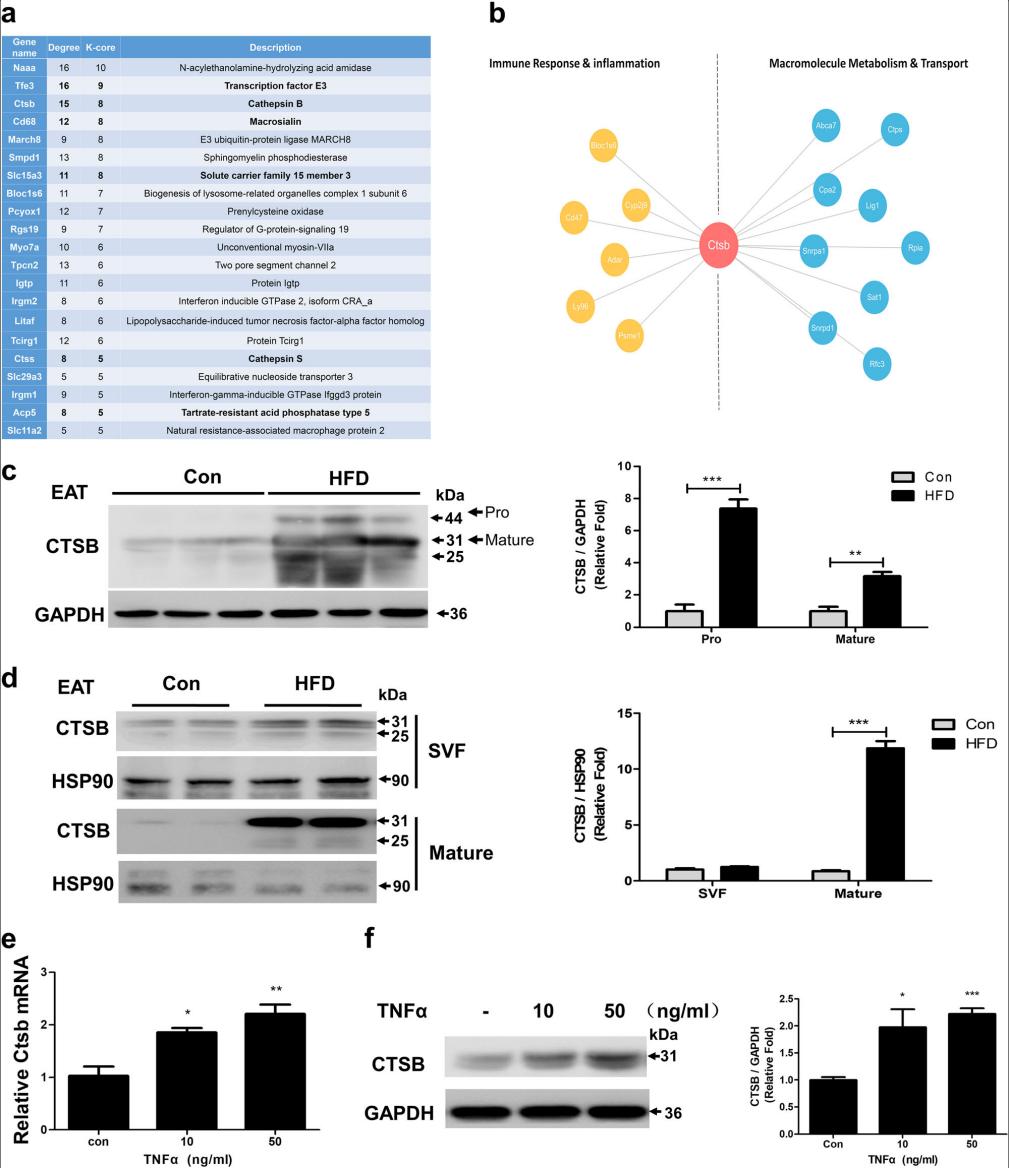
Cathepsin B was identified in the pathogenesis of obesity-related inflammation in adipocytes **a** K-core and degree of lysosomal/autophagic genes in the co-expression network (Supplementary Figure. 2A). **b** Co-expression network of CTSB and its interacting genes. **c–d** C57BL/6 mice were fed a HFD or standard chow for 24 weeks, then expression levels of CTSB in EAT were determined **c** (*n* = 6), along with the SVF and mature adipocytes from EAT via western blot **d**, *n* = 2 for each group, and three mice were pulled together for each sample. 3T3-L1 adipocytes were treated with TNFα at the indicated concentrations for 24 h, then CTSB expression was detected by qRT-PCR **e** and western blot **f**. *p < 0.05, **p < 0.01

Next, we constructed a co-expression network to identify the core lysosomal/autophagic gene(s) with pivotal roles in adipocytes for the response to TNFα treatment (Supplementary Figure. 4A). We applied the “k-core” scores to identify the “key regulatory” genes and found that CTSB (Cathepsin B), a lysosomal cysteine protease primarily involved in the degradation or processing of lysosomal proteins^25^, may play a pivotal role in gene interactions and regulation (Fig. 3a and Supplementary Data File 2). Moreover, co-expression network analysis suggested that CTSB has a connection with the immune response and macromolecule metabolism (Fig. 3b). In HFD-fed mice, CTSB protein was significantly increased in epididymal compared with subcutaneous fat (Fig. 3c, Supplementary Figure. 4B), and the increased protein level was mainly from the mature adipocytes (Fig. 3d). Furthermore, we found that CTSB expression was increased during the differentiation of 3T3-L1 preadipocytes (Supplementary Figure. 4C–D). In differentiated adipocytes, TNFα treatment further upregulated CTSB expression at both the mRNA and protein level (Fig. 3e, f), which was consistent with the results of microarray analysis. We therefore speculate that CTSB may be involved in inflammation-induced autophagy in adipocytes.

### TNFα-induced autophagy in adipocytes required lysosomal proteinase Cathepsin B and was accompanied by transcriptional upregulation of SQSTM1

**Fig. 4:**
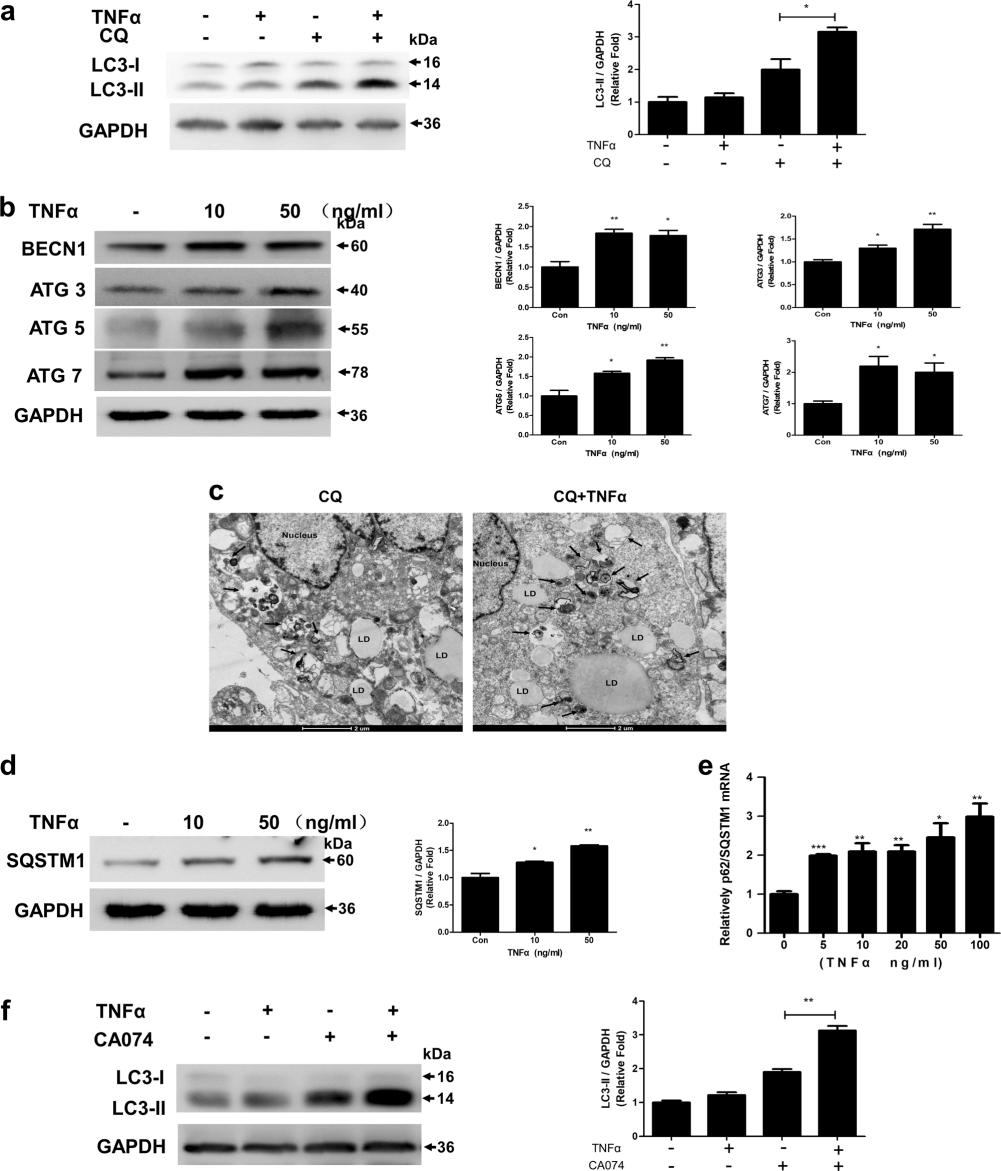
TNFα transcriptionally upregulated SQSTM1 and increased autophagy flux, which was blocked by a Cathepsin B inhibitor **a** Adipocytes were treated with TNFα (10 ng/ml) for 4 h in the presence or absence of CQ, and the levels of LC3 were detected in cell lysates. **b** Immunoblot detection of Becn1, Atg3, Atg5, and Atg7 in adipocytes treated with TNFα for 4 h. **c** Transmission electron microscopy images of autolysosomes in 3T3-L1 adipocytes with or without TNFα (10ng/ml) stimulation; CQ was added 4 h before fixation. Scale bar: 2 µm. **d–e** The SQSTM1 expression in adipocytes with 4 h TNFα treatment was determined by western blot **d** and qRT-PCR **e**. **f** 3T3-L1 adipocytes were pretreated with 10 µm CA074 for 1 h and then stimulated with TNFα (10 ng/ml) for 4 h. Western blot was performed to analyze the status of LC3 and GAPDH. *p < 0.05, **p < 0.01, ***p < 0.001. The results are representative of at least three independent repeats in the cell experiments

We have demonstrated that autophagy flux is activated when adipocytes are incubated in PLA-CM (Fig. 1h). Therefore, we hypothesized that TNFα could activate autophagic flux at a certain time of stimulation. Indeed, 4 h of TNFα stimulation significantly increased the levels of LC3-II in the presence of CQ (Fig. 4a). Consistent with autophagic flux, the expression of autophagy core proteins, such as BECN1, ATG3, ATG5, and ATG7 was also increased (Fig. 4b). Transmission electron microscopy images showed that there were significant increases in the number of autolysosomes in response to TNFα (Fig. 4c). However, when the exposure time was extended to 24 h, autophagic flux and autophagic core protein expression returned to basal levels (Supplementary Figure. 5A). SQSTM1, an autophagy substrate, is frequently used to assess autophagic flux. However, we observed a TNFα dose-dependent increase in SQSTM1 protein expression (Fig. 4d), and SQSTM1 levels further increased in the presence of both TNFα and CQ/bafilomycin (Supplementary Figure. 5B). We further examined SQSTM1 mRNA expression and found that TNFα induces SQSTM1 expression at the transcriptional level at 4 h (Fig. 4e), which recovered at 24 h (Supplementary Figure. 5C). It has been reported that cathepsins have a crucial role in the degradation of sequestered/delivered material during the last step of autophagy^26^. In our experiment, CTSB inhibitor CA074 could block TNFα induced autophagy activity to a similar extend as CQ (Fig. 4f), suggesting CTSB was required for the process of autophagy activity.

Although previous studies demonstrated that CTSB contributes to TNFα-mediated hepatocyte apoptosis^27,28^, we found that CTSB overexpression had no effect on cleaved Caspase 3 (Supplementary Figure. 5D), and the CTSB inhibitor CA074 could not rescue TNFα-induced apoptosis in adipocytes (Supplementary Figure. 5E). CTSB overexpression in 3T3-L1 cells did not affect adipocyte differentiation, as reflected in the expression levels of PPARγ, C/EBPα, Fabp4, Scd1m, and Plin1 (Supplementary Figure. 5F).

### TNFα caused the degradation of PLIN1, which required Cathepsin B

**Fig. 5:**
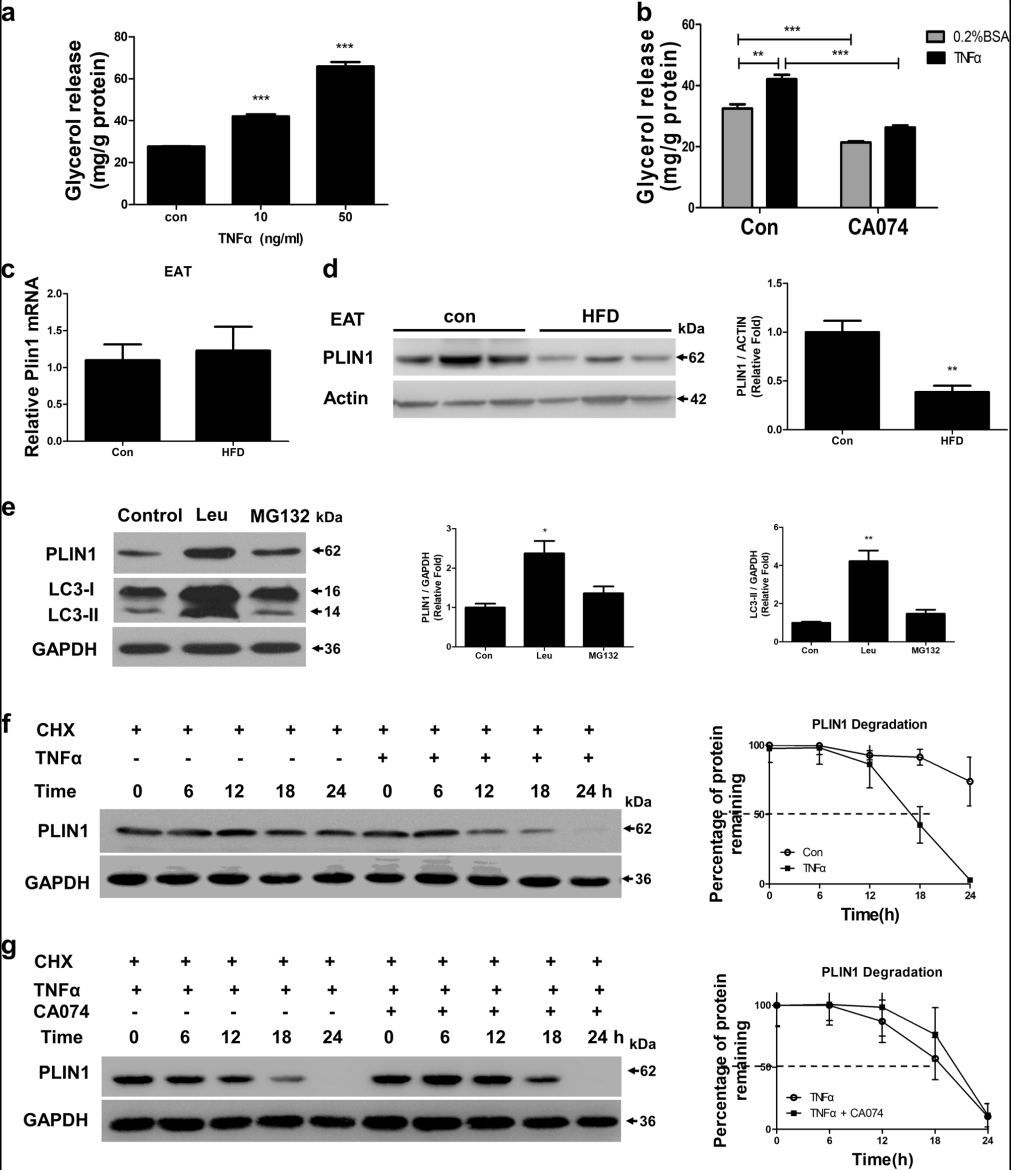
TNFα increased basal lipolysis and degraded PLIN1, which requires Cathepsin B **a** 3T3-L1 adipocytes were individually treated with 10 ng/ml and 50 ng/ml TNFα for 3 h, then media was removed for glycerol measurement. **b** 3T3-L1 adipocytes were stimulated with 10 ng/ml TNFα for 3 h with or without 1 h CA074 pre-treatment, then media was removed for glycerol measurement. **c–d** C57BL/6 mice were fed a HFD or standard chow for 24 weeks, then expression levels of PLIN1 in EAT were determined by qRT-PCR **c** and western blot **d** (*n* = 6). **e** 3T3-L1 adipocytes were treated with the proteasome inhibitor MG-132 (10 μm) or the lysosomal protease inhibitor leupeptin (Leu; 10 μg/ml) for 24 h, then the content of PLIN1 were assessed by western blot. **f** PLIN1 stability in response to TNFα (10 ng/ml) was evaluated in a cycloheximide (CHX, 100 μg/ml) chasing experiment. Immunoblot of perilipin after 6, 12, 18, and 24 h treatments with CHX in adipocytes. **g** Adipocytes were stimulated with TNFα (10 ng/ml) in the presence or absence of CA074 treatment (10 µm). Immunoblotting of PLIN1 was perform after treatment with CHX for 6, 12, 18, and 24 h. **p < 0.01, ***p < 0.001. The results are representative of at least three independent repeats in the cell experiments

We wanted to identify whether TNFα-stimulating autophagy is involved in increased adipocyte lipolysis. Indeed, basal lipolysis was increased by TNFα (Fig. 5a). Moreover, CA074 pre-treatment could partially reverse the effects of TNFα on lipolysis (Fig. 5b). It has been demonstrated that TNFα regulates lipolysis, in part, by decreasing PLIN1 content in adipocytes^29^. Here, we found PLIN1 protein content significantly decreased without being transcriptionally upregulated in the epididymal fat of HFD mice (Fig. 5c, d). It has been reported that both the lysosome and proteasome system may regulate PLIN1 proteolysis^30,31^. In our studies, the PLIN1 content was significantly increased by the lysosomal protease inhibitor leupeptin, suggesting that the lysosomal pathway is primarily responsible for PLIN1 degradation (Fig. 5e). We further verified that TNFα treatment could accelerate the degradation of PLIN1 in adipocytes when co-treated with cycloheximide (CHX) to inhibit protein synthesis (Fig. 5f). However, blockage of CTSB activity with CA074 delayed TNFα-mediated degradation of PLIN1 (Fig. 5g). These results suggested that TNFα-induced degradation of PLIN1 through lysosomal pathway and Cathepsin B might play a role in the obesity process.

### The autophagy–lysosome pathway mediates the degradation of PLIN1

**Fig. 6:**
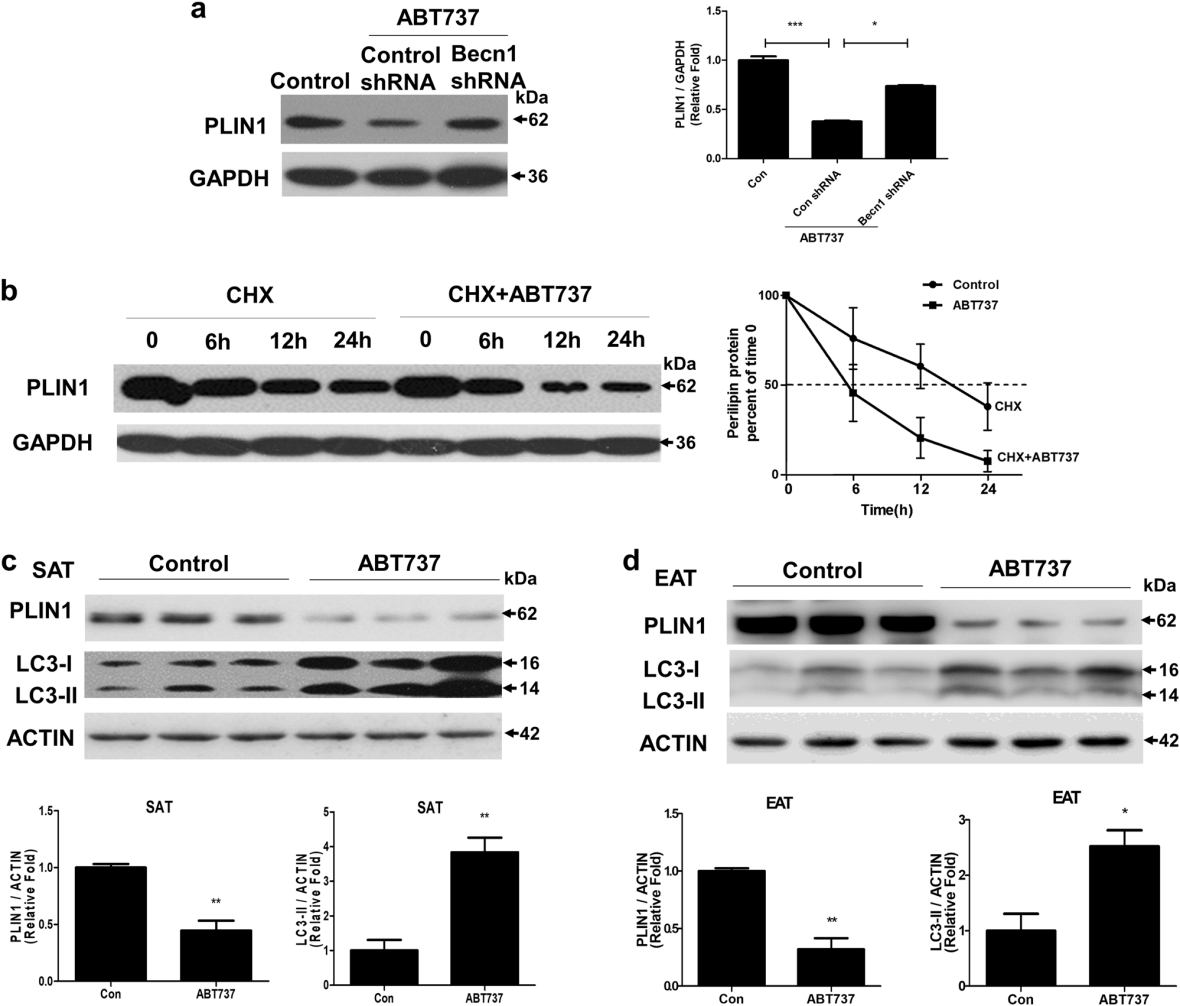
PLIN1 degradation could be accelerated by the activation of the autophagy–lysosome pathway **a** Mature adipocytes infected with lentivirus-mediated control shRNA or shRNA to Becn1 were treated with ABT737 (100 nm) for 12 h. Representative immunoblots of PLIN1 and GAPDH. **b** Cells were harvested at 0, 6, 12, or 24 h after the addition of CHX (100 μg/ml) with or without ABT737 treatment (100 nm), then the stability of PLIN1 was evaluated using western blot. **c**–**d** 8-week-old male C57BL/6 mice were injected intraperitoneally with ABT737 (75 mg/kg) for 14 consecutive days (*n* = 6), then protein expression of PLIN1 and LC3 in the subcutaneous fat **c** and epididymal fat **d** was determined by western blot. *p < 0.05, **p < 0.01, ***p < 0.001. The results are representative of at least three independent repeats in the cell experiments

Our previous study has found ABT737, a BH3 mimetic that modulates the Bcl-2-Beclin1 interaction, can induce autophagy in adipocytes^20^. Next, we wanted to explore whether the autophagy activity induced by ABT737 could accelerate PLIN1 degradation, and found ABT737 decreased PLIN1 protein levels, which could be recovered by lenti-shBecn1 (Fig. 6a). In addition, ABT737 led to PLIN1 instability and promoted its degradation (Fig. 6b). These results indicate that the autophagy–lysosome pathway is involved in PLIN1 degradation. Furthermore, to verify whether these ABT737 effects on PLIN1 were consistent in vivo, 8-week-old male C57BL/6 mice were intraperitoneally injected with ABT737 for 14 days, then subcutaneous fat and epididymal fat was isolated for Western blot assays. As shown in Fig. 6c, d, ABT737 significantly decreased the PLIN1 content in both the subcutaneous and epididymal fat. At the same time, ABT737 increased LC3-II levels. Thus, these results suggested that PLIN1 is degraded through the autophagy–lysosome pathway in adipose tissue.

### The selective autophagy receptor SQSTM1-mediated degradation of PLIN1 via ubiquitin-recognition machinery

**Fig. 7:**
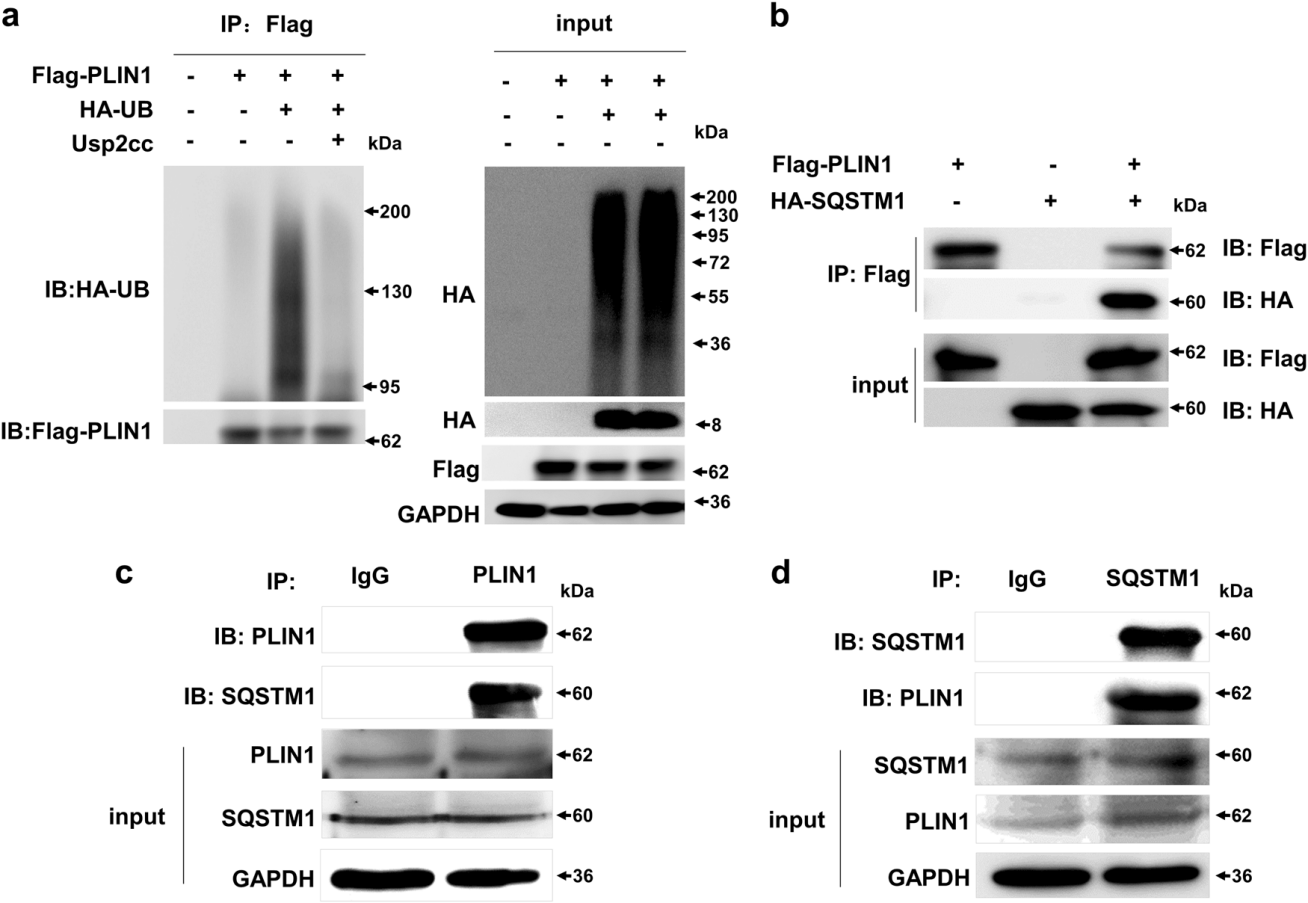
PLIN1 can be ubiquitylated and directly interacts with SQSTM1 **a** 293T cells were transfected with the Flag-PLIN1 plasmid and/or HA-UB plasmid with or without usp2cc (the catalytic core of human ubiquitin-specific protease 2) treatment, followed by immunoblotting with anti-HA and anti-Flag. **b** 293T cells were transfected with the Flag-PLIN1 plasmid and/or HA-SQSTM1 plasmid as indicated, and the lysates were immunoprecipitated with flag beads, then immunoblotted with the indicated antibodies. **c** In 3T3-L1 adipocytes, the lysates were immunoprecipitated with anti-PLIN1, then immunoblotted with the indicated antibodies. **d** In 3T3-L1 adipocytes, the lysates were immunoprecipitated with anti-SQSTM1, then immunoblotted with the indicated antibodies

According to the preceding results, TNFα can upregulate SQSTM1 expression at the transcriptional level. It has been reported that SQSTM1 is a cargo receptor for the recognition and degradation of ubiquitinated substrates^32^. We postulated that SQSTM1 may have a role in the autophagic degradation of PLIN1. As expected, ectogenous PLIN1 was found to be ubiquitinated in 293T cells (Fig. 7a). We then examined the interaction between PLIN1 and SQSTM1. We performed co-immunoprecipitation experiments in 293T cells that expressed a HA-tagged plasmid of SQSTM1 and a Flag-tagged plasmid of PLIN1; we determined that PLIN1 interacts directly with SQSTM1 (Fig. 7b). Moreover, the interaction between endogenous PLIN1 and SQSTM1 is also detectable in mature adipocytes (Fig. 7c, d). Thus, PLIN1 can be ubiquitinated and recognized by SQSTM1, indicating a selective autophagy mechanism mediated degradation of PLIN1.

## Discussion

Adipose tissue stores excess energy in the form of lipids and is thus able to markedly change its mass in response to metabolic stress. Obese adipocytes exhibit high metabolic activity along with an increase in lipid mobilization. Autophagy is a critical regulator of metabolic homeostasis, but its activity and role in the adipocytes of obese individuals remains unclear. In this study, we demonstrate that 34 lysosomal/autophagic genes are significantly upregulated in the omental adipose tissue of obese individuals, which contributes to an increase in autophagy activity in the adipocytes. The proinflammatory cytokines secreted by macrophages account for initiation of this process, which occurs in tandem with increased transcription of SQSTM1, suggestive of a selective autophagy mechanism. PLIN1, an essential protein for lipid storage and lipolysis, can be ubiquitinated and then degraded through SQSTM1-mediated autophagy under obesity-related inflammatory states. Thus, this study supports the notion that autophagy plays a crucial role in inflammatory cytokine-mediated lipid mobilization in obesity.

Recent studies indicate that key autophagy genes, LC3A, LC3B, and Atg5, are elevated in the adipose tissue of obese individuals, suggesting a transcriptional regulatory mechanism^13^. Here, we analyze the expression behavior of a previously reported set of 322 lysosomal/autophagic genes^19^ in our RNA-seq data. Thirty-five differentially expressed genes were detected, 34 of which were upregulated. This finding raises two questions. What factors drive an increased lysosomal/autophagic genes expression? Is it adipocytes or SVF cells within the fat that contribute to changes in the expression of these genes? As obesity is associated with a state of low-grade inflammation, we speculate that the overproduction of inflammatory cytokines in obese adipose tissue may transcriptionally upregulate lysosomal/autophagic genes in adipocytes. We therefore analyzed a data set of TNFα-induced genes in 3T3-L1 adipocytes^24^ and found that TNFα upregulated the expression of lysosomal/autophagic genes within 24 h.

The majority of the reports regarding increased expression of a variety of autophagy-related genes are consistent with an elevated autophagic flux^33–35^. However, in adipocytes, it is still controversial whether this process actually drives autophagy. We used differentiated 3T3-L1 adipocytes to evaluate autophagy flux and found that the autophagy flux in adipocytes is increased when exposed to PLA-CM. Also, TNFα treatment alone resulted in an elevated autophagy flux. These results suggest that in obesity, the expression of the autophagic machinery components in adipose tissue is upregulated by inflammatory cytokines and reflects increased autophagic flux in adipocytes. On the other hand, prolonged inflammatory stress can lead to an overall downregulation of autophagy-related genes and may ultimately impair autophagy/lysosomal function. However, either too little or too much autophagy is a pathological process that can contribute to adipocyte dysfunction.

Previous studies used adipose tissue explants and adipocytes as models for autophagy activity assessment^10,36–38^. Kovsan J et al. first reported an elevated autophagic flux in fat explants from obese individuals using SQSTM1/p62 and a LC3 turnover assay and lysosomal inhibitor. Soussi H et al. showed the autophagic substrate SQSTM1/p62 was elevated in obese vs. lean adipocytes in both protein and mRNA levels, precluding any definite conclusion on autophagic flux based on SQSTM1/p62 protein content. Soussi H et al. further demonstrated an attenuated, rather than activated, autophagic flux in adipocytes from obese individuals using a LC3 turnover assay and lysosomal inhibitor. Moreover, Mizunoe et al. showed that autophagosome formation was accelerated in WAT explants measuring by CQ and LC3 turnover assay in HFD mice, which was in accordance with Kovsan J et al. and opposite to Soussi H et al. However, they considered autophagic clearance was impaired using a SQSTM1/p62 turnover index under rapamycin stimulation. It should be considered that autophagy is highly stress sensitive and can be strongly induced in the interval between the environmental change and the cell adaptation. The interferences of the isolation process and a drastic change in environmental conditions may result in the inconsistency of various studies. According to the guideline^3^, transmission electron microscopy (TEM) is the most accurate method for the detection of autophagy. However, all of the studies, including ours, did not dynamically monitor the whole process of autophagy by TEM. Because the lipid droplets in mature adipocytes make it difficult to observe the organelles, it is important and urgent to develop the morphological observation method for the autophagic process by TEM in further research.

Although SQSTM1, as an autophagy substrate, is widely used as an indicator of autophagy flux in various tissues and cells, including adipose tissue and adipocytes^36,37^. Here, we show that SQSTM1 can be transcriptionally upregulated by obesity-related inflammatory stimuli in mature adipocytes, which indicated autophagy activity might be underestimated in obese conditions when SQSTM1 was used as an autophagy substrate. It is noteworthy that SQSTM1 is vital in the selective autophagy of ubiquitinated proteins. Here, we verify PLIN1 can be ubiquitinated via the co-transfection of Flag-PLIN1 and HA-UB, with or without the catalytic core domain of the USP2 deubiquitinase. Given that PLIN1 is a highly stable protein under basal conditions, the proteolysis pathway seems to be required for altering the PLIN1 content to regulate lipolysis. As previously reported, lipid droplets are surrounded by structural proteins of the PLIN family, with PLIN1 being primarily an adipocyte protein, whereas PLIN2 and PLIN3 are expressed ubiquitously^39^. Ana Maria Cuervo et al.^40^ reported that PLIN2 and PLIN3 are chaperone-mediated autophagy (CMA) substrates and their degradation through CMA precedes lipolysis. However, the degradation pathway of PLIN1 has not been clearly reported.

Furthermore, we verified that TNFα has an effect on the degradation of PLIN1 by SQSTM1-mediated selective autophagy. Therefore, it is important to consider the possibility that autophagy may play a key role in inflammation-related lipid metabolism in adipose tissue under obese conditions. Adipose tissue is a triglyceride reservoir and a buffer to lipid flux, in which inflammatory stress-induced autophagy exacerbates lipolysis and ectopic lipid deposition, then contributes to systemic insulin resistance.

It is notable that the lysosome is the final destination of autophagy-lysosomal degradation^41,42^. Lysosomal dysfunction is the most common cause of many diseases associated with autophagy disorders, such as cancer and neurodegenerative diseases^43–45^. Degradation in lysosomes occurs through the concerted action of > 50 soluble acid hydrolases. We found that among all the genes differentially expressed between lean and obese human adipose tissue, lysosomal hydrolases seem to be the most highly upregulated, emphasizing the importance of lysosomal function for lipid metabolism homeostasis. We further identified the expression levels of CTSB and CTSS, which were upregulated in TNFα-treated adipocytes. It has been reported that CTSB is required for autophagic flux and autophagic degradation^46,47^. Therefore, further studies should focus on the alteration of lysosomal function in autophagic homeostasis of adipocytes.

**Fig. 8:**
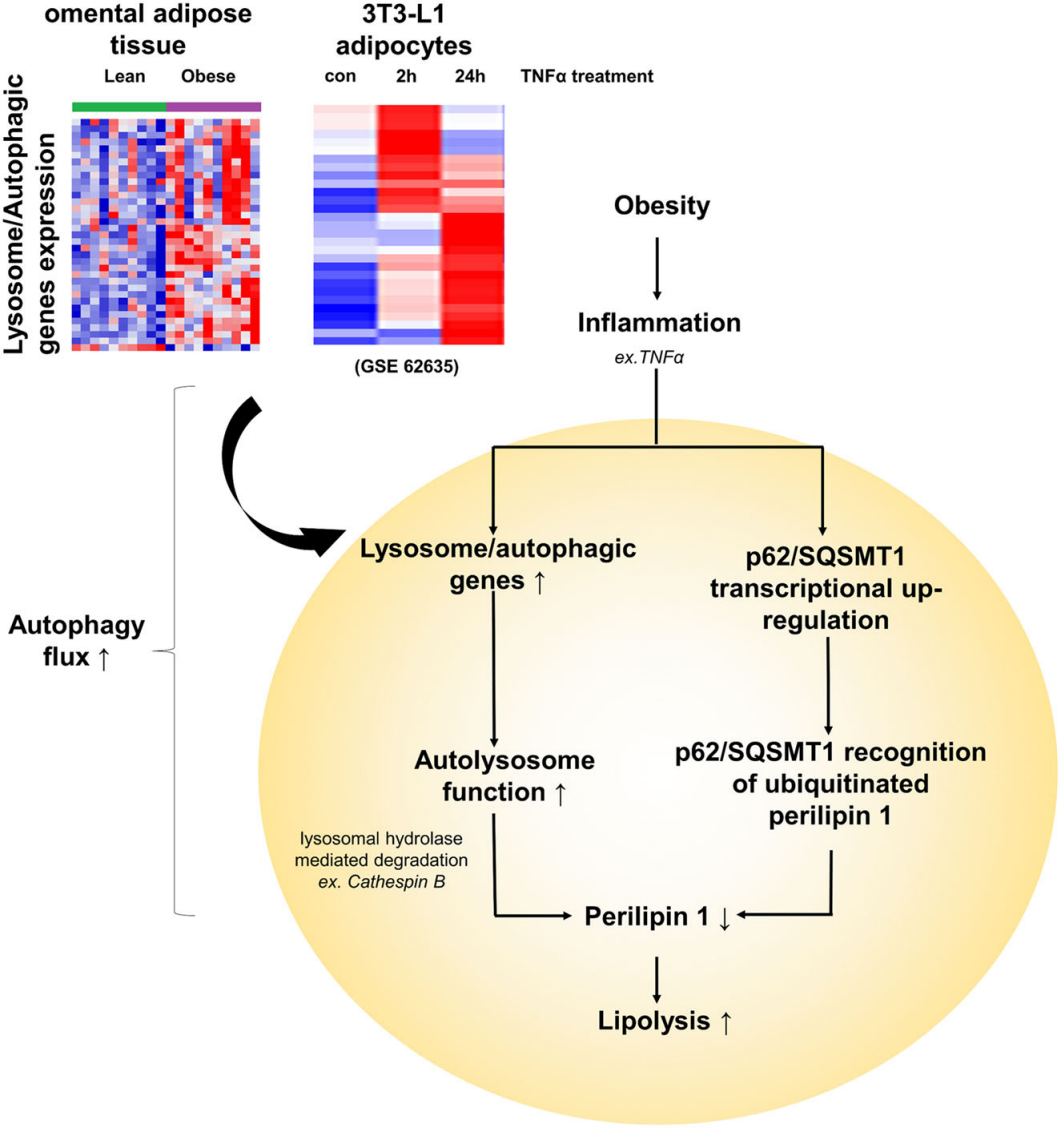
Schematic of the autophagy–lysosomal response to obesity-associated adipose inflammation in adipocytes In obese individuals, we specifically analyzed the expression changes of 322 lysosomal/autophagic genes, and identified 34 upregulated genes among 35 altered genes. In mature adipocytes, proinflammatory TNFα also caused a major upregulation of all changed lysosomal/autophagic genes within 24 h and an overall downregulation tendency under chronic stimulation, which revealed impaired autophagic function. Short term TNFα treatment led to an increase in autophagic flux, accompanied by transcriptional upregulation of SQSTM1, suggesting the existence of SQSTM1-mediated selective autophagy. Increased SQSTM1 contributed to autophagic degradation of PLIN1, a master regulator of lipolysis. As autophagy and lipolysis share similarities in regulation and function, our findings showed that a moderate inflammation in local adipose tissue may link autophagy to lipolysis under obese conditions

Overall, we observed a significant upregulation of lysosomal/autophagic genes, both in the omental adipose tissue of obese individuals and in adipocytes challenged with TNFα. This is in accordance with an increased autophagy flux in adipocytes. TNFα-induced autophagy is a more selective process that is facilitated by SQSTM1 to selectively degrade PLIN1 (Fig. 8). Our study describes links between inflammation-related autophagy and lipid metabolism in adipose tissue. We have also provided new insights into the progressive lysosomal dysfunction as a crucial mechanism involved in the obesity process.

## Methods

### Human subjects

From January to December 2015, we recruited 11 subjects with a BMI between 18.5 and 24.9 as lean controls, and 10 severely obese subjects with a BMI between 34 and 56, all without diabetes, coronary heart disease, liver dysfunction, kidney disease, cancer, or any other chronic diseases, who underwent a laparoscopic cholecystectomy in Shanghai Jiao Tong University Affiliated Sixth People's Hospital (Shanghai, China) and Shanghai Seventh People's Hospital (Shanghai, China). Wedge biopsies of visceral adipose tissue (omentum majus) and subcutaneous adipose tissue (abdominal) were collected via a laparoscope during the operation and stored at − 80 °C. This study was approved by the Human Research Ethics Committee of Shanghai Jiao Tong University Affiliated Sixth People's Hospital and Shanghai Seventh People's Hospital. Written informed consent was obtained from each subject.

### Animals

C57BL/6 male mice were purchased from the Shanghai SLAC Laboratory Animal Company. HFD-induced obese mice were maintained with free access to high-fat chow (60% fat, Research Diets, D12492). All mice were free to drink water and housed at 21 °C ± 1 °C with a humidity of 55% ± 10% and a 12-hour light/dark cycle. For autophagy activator treatment, ABT737 (Selleck, S1002) was intraperitoneally injected at a dose of 75 mg/kg daily in for 14 days. All animal procedures were approved by the Animal Care Committee of Shanghai Jiaotong University School of Medicine.

### Cells

3T3-L1 and 293T cell lines were purchased from the American Cell Type Culture Collection (ATCC, USA). The 3T3-L1 cell line was cultured in DMEM (GIBCO, 11995) containing 10% fetal bovine serum (FBS, GIBCO, 10099–141). We induced commitment to the adipocyte lineage as described previously^48^. 293T cells were cultured in 10% FBS-DMEM. For immunoprecipitation, ~ 1.0 × 10^6^ cells were plated into each well of a six-well plate 24 h before transfection. Plasmid (800 ng/well) transfections were performed using Lipofectamine 2000 (Invitrogen, 11668), according to the manufacturer’s protocol.

### Preparation of CM and cell treatments

Macrophage-CM of bone marrow-derived macrophages (BMDMs) was provided by Professor Weili Shen and made as described^49^. In brief, the CM was divided into two groups: BMDMs from C57BL/6 mice without stimulation (Control-CM) and those stimulated with 0.5 mm palmitate for 16 h (PLA-CM). To simulate inflammatory conditions in adipocytes, 3T3-L1 adipocytes were placed either in Control-CM or PLA-CM for 24 h. TNFα (Sangon Biotech, C600052) was used as a stimulating factor of inflammation in 3T3-L1 adipocytes. CQ (Sigma, C6628) and Leupeptin (Sangon Biotech, A600580) were used as lysosomal inhibitors. MG-132 (APExBIO, A2585) was used as a proteasome inhibitor. ABT737 (Selleck, S1002), a BH3 mimetic, was used to induce autophagy. CA074 (APExBIO, A1926), a Cathepsin B inhibitor was applied for one hour prior to drug stimulation at a concentration of 10 µm. To determine the stability of PLIN1 in 3T3-L1 adipocytes, on day 8 after induction, cells were treated with cycloheximide (CHX) (Amresco, 94271) and harvested at different time points after the addition of CHX.

### Transcriptome analysis for human adipose tissue

Paired-end libraries were synthesized using the TruSeq® RNA Sample Preparation Kit (Illumina, RS-122-2001) following the supplied guidelines. Library construction and Illumina sequencing was performed at the Shanghai Biotechnology Corporation. High quality reads that passed the Illumina quality filters were kept for sequence analysis, and bioinformatic data analysis was performed by the Shanghai Novel Bioinformatics Company.

### RNA-sequencing and data treatment

Raw reads were obtained after sequencing and filtered with the adaptor to remove low quality reads and contaminated sequences to achieve clean data. Clean data was mapped to the human hg19 genome utilizing Tophat2^50^, and fragments were calculated by HTSeq^51^. Genes were considered significantly differentially expressed under the following criteria using DESeq:^18^ 1) Fold change > 1.2 or < 0.833 and 2) FDR < 0.2.

### Co-expression analysis

We used gene co-expression networks to find the relationships among genes^52^. Gene co-expression networks were built according to the normalized expression values of genes selected from genes in functional analysis. For each pair of genes, we calculated the Pearson correlation coefficient and chose the significant correlation pairs (FDR < 0.05) to construct the network^53^.

### Microarray data analysis

Microarray data related to gene expression changes in 3T3-L1 adipocytes caused by TNFα in a time-dependent manner^24^ were analyzed here, which can be accessed at the NCBI GEO database (http://www.ncbi.nlm.nih.gov/geo/) (GSE62635). We applied limma package^54^ to filter the differentially expressed genes, after the significant analysis under the following criteria: 1) Fold Change > 2 or < 0.5 and 2) P-value < 0.05. We selected the differentially expressed genes between time points 0 h, 2 h, 24 h, and 6 d. In accordance with the different RPKM change tendencies of genes under different situations, we identified a set of unique model expression tendencies. Using a strategy for clustering time-series gene expression data, we defined some unique profiles. The expression model profiles are related to the actual or the expected number of genes assigned to each model profile. Significant profiles have a higher probability than expected by Fisher’s exact test and multiple comparison tests^55^.

### Functional annotation analysis

Gene ontology (GO) analysis was performed to facilitate elucidating the biological implications of unique genes in the significant or representative profiles of the differentially expressed genes^56^. We downloaded the GO annotations from NCBI (http://www.ncbi.nlm.nih.gov/), UniProt (http://www.uniprot.org/), and the Gene Ontology (http://www.geneontology.org/). Fisher’s exact test was applied to identify the significant GO categories, and FDR was used to correct the P-values. Pathway analysis was used to find significantly altered pathways containing differentially expressed genes according to the KEGG database. We used the Fisher’s exact test to identify significant pathways, and the threshold of significance was defined by P-value and FDR^57^.

### TEM

3T3-L1 adipocytes were washed with phosphate buffer saline (PBS) and fixed in 2.5% glutaraldehyde in PBS for 2 h. Then, cells were washed three times with 0.1 m phosphate buffer and fixed with 1% osmium tetroxide in 0.1 m phosphate buffer for another 2 h. Samples were dehydrated with increasing concentrations of ethanol and 100% acetone, then embedded in epoxy resin. Electron photomicrographs were taken from the ultrastructures of 3T3-L1 adipocytes under a transmission electron microscope (JEM-1200EX, Japan).

### TG measurement

Mature 3T3-L1 adipocytes were first treated with TNFα in serum-free DMEM containing 0.2% bovine serum albumin (BSA) for 24 h, then incubated with serum-free DMEM containing 0.2% BSA for 3 h. The glycerol content in the incubation medium was used as an index for lipolysis and measured using GPO-Trinder reagent (Sigma, FG0100) based on the manufacturer’s instructions.

### Lentivirus transduction

A lentivirus containing the CTSB expression vector was purchased from the Shanghai GeneChem Corporation. Lentivirus-mediated control shRNA and shRNA to Becn1 was ordered from the Shanghai Genepharma Corporation. The virus was used at a multiplicity of infection (MOI) of 10 to infect 3T3-L1 preadipocytes and MOI of 50 to infect 3T3-L1 adipocytes. The efficiency of infection was assessed by qRT-PCR and Western blot analysis.

### Western blotting

Cultured cells or the subcutaneous and epididymal adipose tissues of mice were lysed in radioimmunoprecipitation buffer (Beyotime Biotechnology, P0013B) containing protease and phosphatase inhibitors. Lysates were centrifuged at 13,000 g for 30 min at 4 °C. Protein concentrations of the extracts were determined with a Pierce BCA Protein Assay Kit (Thermo, 23227). Membranes were incubated with GAPDH (KangChen Biotech, KC-5G4), HSP90 (Cell Signaling Technology, 4877), ACTIN (Cell Signaling Technology, 4967), Cathepsin B (CTSB) (Cell Signaling Technology, 31718), PLIN1 (Cell Signaling Technology, CST, 9349s), SQSTM1 (Cell Signaling Technology, 5114), and autophagy antibodies (Cell Signaling Technology, 4445), including BECN1, LC3A/B, ATG5, ATG16L1, ATG7, and ATG3 overnight at 4 °C. The membranes were incubated with an appropriate secondary antibody conjugated with horseradish peroxidase for 1 h at room temperature. ECL Prime Western blotting Detection Reagent (GE Healthcare, RPN2232) was used to visualize protein bands by electro-chemoluminescence (ImageQuant LAS4000, USA).

### Immunoprecipitation assays

293T cells were transfected with Flag-PLIN1 and HA-UB plasmids (provided by Hu. lab). Ubiquitylation of ectogenic PLIN1 in 293T cells was assessed by immunoprecipitation (IP) using anti-Flag antibodies, with or without usp2cc (the catalytic core of human ubiquitin-specific protease 2, provided by Hu. lab) and followed by immunoblotting with anti-HA and anti-Flag. During in vitro co-immunoprecipitation, 293T cells were transfected with Flag-PLIN1 and HA-SQSTM1 plasmids and harvested 48 h later. For in vivo co-immunoprecipitation, endogenous PLIN1 and SQSTM1 was immunoprecipitated from 3T3-L1 adipocytes. Cells were resuspended in lysis buffer (50 mm Tris-HCl pH 7.4, 150 mm NaCl, 1 mm ethylenediaminetetraacetic acid, 1% TRITON X-100) containing protease and phosphatase inhibitors. The lysates were immunoprecipitated with anti-Flag (Sigma, A2220), protein A/G plus beads (Santa Cruz, sc-2003), and anti-PLIN1 or anti-SQSTM1 at 4 °C overnight and then immunoblotted with anti-HA (Santa Cruze, sc-7392) and anti-SQSTM1 or anti-PLIN1.

### RNA isolation and real-time PCR

Total RNA was isolated using the Trizol reagent (Invitrogen, 15596018) and converted into complementary DNA (cDNA) using the iScript cDNA Synthesis Kit (Bio-Rad, 170–8891). Quantitative PCR analysis was performed using SYBR Green Premix Ex Taq (Takara) on a Light Cycler 480 (Roche). The murine 36b4 gene served as the internal control. Evaluation of the relative differences in the PCR products of the treatment groups was performed using the △△CT method. The reciprocal of 2CT (the CT was used as an exponent for the base 2) for each target gene was normalized to that of the 36b4 gene. The sequences of primers used are presented in Supplementary Table 3.

### Statistical analysis

Clinical basic data in line with the normal distribution is presented as mean ± standard deviation, whereas the partial distribution of data is presented as the median (four point spacing). Other data were presented as mean ± the standard error of the mean. Statistical differences were calculated using a Student’s *t* test or Mann–Whitney *U* test. Significance was shown as ∗*p* < 0.05, ∗∗*p* < 0.01 or ∗∗∗*p* < 0.001.

## Supplementary information

Supplementary information

Figure S1

Figure S2

Figure S3

Figure S4

Figure S5

Supplementary Data File 1

Supplementary Data File 2
