# Supplementary material for: Obesity-associated inflammation triggers an autophagy–lysosomal response in adipocytes and causes degradation of perilipin 1
Source: Cell Death Dis. 2019 Feb 11;10(2):121. doi: 10.1038/s41419-019-1393-8 (PMC6370809; doi:10.1038/s41419-019-1393-8)
Supplement: Supplementary file 1 — Supplementary information [file 41419_2019_1393_MOESM1_ESM.docx]

**Obesity-associated** **inflammation triggers an autophagy-lysosomal response in adipocytes and causes degradation of perilipin1**

Running title: Inflammation related autophagy mediates lipolysis

Liping Ju^1*^, Junfeng Han^1*^, Xiaoyan Zhang^2,3^, Yujie Deng^2,4^, Han Yan^1^, Congrong Wang^1^, Xiaohua Li^5^, Shuqin Chen^1^, Miriayi Alimujiang^1^, Xu Li^1^, Qichen Fang^1^, Ying Yang^1#^, Weiping Jia^1#^

**This Supplementary information includes:**

**Supplementary Table: Table 1-3**

**Supplementary Figure: Figure 1-5**

**Supplementary References**

**Supplementary Table 1: Clinical data baseline**

|  | **normal** | **obesity** | ***P* value** |
| --- | --- | --- | --- |
| n | 11 | 10 |  |
| Male/Female | 3/8 | 4/6 |  |
| Age, years | 42.5 + 9.4 | 28.2 + 7.8 | 0.002 |
| BMI, kg/m^2^ | 23.7 + 4.0 | 41.8 + 7.7 | < 0.001 |
| FPG, mmol/L | 5.0 + 0.48 | 5.5 + 0.49 | 0.07 |
| SBP, mmHg  DBP, mmHg  ALT, U/L  AST, U/L  T-BIL, μmol/L | 120.4 + 10.3  79.9 + 7.3  15（11.5-17.1）  17（14-19）  10.8（9.7-14.0） | 125.8 + 21.0  81.9 + 9.1  16.5（13.8-25.8）  16（15-21）  9.8（7.5-11.1） | 0.48  0.60  0.39  0.84  0.08 |
| D-BIL, μmol/L | 3.8（2.1-4.8） | 3.3（3.2-5.1） | 0.57 |
| Scr, μmol/L | 57.2（47.4076） | 64.5（53.8-72.3） | 0.45 |
| BUN, mmol/L | 4.2（3.2-4.8） | 4.5（3.9-4.8） | 0.46 |

Abbreviations: BMI, body mass index; FPG, fasting plasma glucose; SBP, systolic blood pressure; DBP, diastolic blood pressure; ALT, alanine aminotransferase; AST, aspartate aminotransferase; T-BIL, total bilirubin; D-BIL, direct bilirubin; Scr, serum creatinine; BUN, blood urea nitrogen.

Continuous variables are displayed as means ± standard deviations (SDs) or medians (interquartile ranges) and categorical variables as numbers. Differences were assessed using Student's t test or Mann-Whitney U test (for continuous variables).

**Supplementary Table 2 : 35 lysosome / autophagic genes**

| **Gene** | **Ortholog** | **Function** | **Location** | **References** |
| --- | --- | --- | --- | --- |
| ABCA2 | Mouse, all | Transporter | Lysosome, lysosomal membrane, endosome | [[1](#_ENREF_1)] |
| ACP5 | Mouse, all | Lysosomal acid phosphatases | Extracellular, cytosol, lysosome | [[2](#_ENREF_2)] |
| ADRB2 | Mouse, all | Signal transduction | Plasma membrane, lysosome, endosome | [[3](#_ENREF_3)] |
| ARSB | Mouse, all | Lysosomal enzyme | Extracellular, endoplasmic reticulum, lysosome | [[4](#_ENREF_4)] |
| ASAH1 | Mouse, all | Lysosomal hydrolase | Extracellular, lysosome | [[5](#_ENREF_5)] |
| CD68 | Mouse, all | Scavenger receptor,lysosomal/endosomal-associated membrane glycoprotein | Plasma membrane, lysosome, endosome | [[6](#_ENREF_6)] |
| CHIT1 | Mouse, all | Chitotriosidase | Extracellular, lysosome | [[7](#_ENREF_7)] |
| CLN8 | Mouse, all | Transmembrane protein | Endoplasmic reticulum | [[8](#_ENREF_8)] |
| CREG1 | Mouse, all | Lysosomal protein | Extracellular, lysosome | [[9](#_ENREF_9)] |
| CTSB | Mouse, all | Lysosomal cysteine protease | Extracellular, nucleus, lysosome, endosome | [[10](#_ENREF_10), [11](#_ENREF_11)] |
| CTSS | Mouse, all | Lysosomal cysteine proteinase | Extracellular, lysosome, endosome | [[12](#_ENREF_12)] |
| EPDR1 | Mouse, all | Transmembrane protein | Extracellular, lysosome | [[13](#_ENREF_13)] |
| FOXO3 | Mouse, all | Transcription factors | Nucleus, cytosol | [[14](#_ENREF_14)] |
| FYCO1 | Mouse, all | Transporter | Lysosome, endosome | [[15](#_ENREF_15), [16](#_ENREF_16)] |
| GAA | Mouse, all | Lysosomal alpha-glucosidase | Plasma membrane,extracellular, lysosome | [[17](#_ENREF_17), [18](#_ENREF_18)] |
| GM2A | Mouse, all | Glycolipid transport protein | Extracellular, lysosome | [[19](#_ENREF_19), [20](#_ENREF_20)] |
| GNS | Mouse, all | Lysosomal enzyme | Extracellular, lysosome | [[21](#_ENREF_21)] |
| GRN | Mouse, all | Secreted glycosylated peptides | Extracellular, lysosome, endosome | [[22](#_ENREF_22)] |
| HYAL2 | Mouse, all | Receptor | Plasma membrane, endoplasmic reticulum, cytosol, lysosome, golgi apparatus | [[23](#_ENREF_23)] |
| LRBA | Mouse, all | Intracellular vesicles transporter | Plasma membrane, cytosol | [[24](#_ENREF_24)] |
| MAPT | Mouse, all | Microtubule-associated protein tau | Plasma membrane, cytoskeleton, nucleus, cytosol | [[25](#_ENREF_25)] |
| MITF | Mouse, all | Transcription factors | Nucleus | [[26](#_ENREF_26), [27](#_ENREF_27)] |
| OSTM1 | Mouse, all | Degradation of proteins | Lysosome, cytosol | [[28](#_ENREF_28)] |
| PINK1 | Mouse, all | A serine/threonine protein kinase that localizes to mitochondria | Mitochondrion, nucleus, cytosol | [[29](#_ENREF_29), [30](#_ENREF_30)] |
| PSAP | Mouse, all | Secretory protein,integral membrane protein | Plasma membrane,extracellular, lysosome | [[31](#_ENREF_31)] |
| SIAE | Mouse, all | Lysosomal enzyme | Extracellular, lysosome | [[32](#_ENREF_32)] |
| SIDT2 | Mouse, all | Transmembrane protein, RNA transporter | Lysosome | [[33](#_ENREF_33)] |
| SLC15A3 | Mouse, all | Lysosomal solute carrier transporters | Lysosome | [[34](#_ENREF_34)] |
| SLC36A1 | Mouse, all | Proton-dependent, small amino acid transporter | Plasma membrane, lysosome | [[35](#_ENREF_35)] |
| STS | Mouse, all | Multi-pass membrane protein, sulfatase | Plasma membrane, endoplasmic reticulum, lysosome | [[36](#_ENREF_36)] |
| STX7 | Mouse, all | Membrane receptor involved in vesicle transport | Plasma membrane,extracellular, lysosome, endosome | [[37](#_ENREF_37)] |
| TFE3 | Mouse, all | Transcription factors | Nucleus, cytosol | [[38](#_ENREF_38)] |
| TFEC | Mouse, all | Transcription factors | Nucleus | [[39](#_ENREF_39), [40](#_ENREF_40)] |
| TM9SF1 | Mouse, all | Transmembrane protein | Plasma membrane, lysosome | [[41](#_ENREF_41)] |
| UVRAG | Mouse, all | A subunit of the class III phosphatidylinositol 3-kinase (PtdIns 3-kinase) complex | Cytoskeleton, endoplasmic reticulum, lysosome, endosome | [[42](#_ENREF_42)] |

**Supplementary Table 3: Primers for Real-time quantitative PCR analysis in mice**

| Genes | Forward | Reverse |
| --- | --- | --- |
| TFE3 | TGCCTGTGTCAGGGAATCTG | CGACGCTCAATTAGGTTGTGAT |
| CD68 | GGAAATGCCACGGTTCATCCA | TGGGGTTCAGTACAGAGATGC |
| CTSS | TGTAGATGCGCGTCATCCTTC | CCAACCACAAGTACACCATGAT |
| CTSB | GAGCTGGTCAACTATGTCAACA | GCTCATGTCCACGTTGTAGAAGT |
| ACP5 | TGAGGACGTATTCTCTGACCG | CACATTGGTCTGTGGGATCTTG |
| SLC15A3 | TGGCGTTTATTCAGCAGAACA | TCTCTGGCCGAGTGTCGTT |
| Tfe3 | TGCGTCAGCAGCTTATGAGG | AGACACGCCAATCACAGAGAT |
| Cd68 | TGTCTGATCTTGCTAGGACCG | GAGAGTAACGGCCTTTTTGTGA |
| Ctss | CCATTGGGATCTCTGGAAGAAAA | TCATGCCCACTTGGTAGGTAT |
| Ctsb | TCCTTGATCCTTCTTTCTTGCC | ACAGTGCCACACAGCTTCTTC |
| Acp5 | CACTCCCACCCTGAGATTTGT | CATCGTCTGCACGGTTCTG |
| Slc15a3 | GAACGCGCTGCCTTCTTTG | GTCCCAGGTACACGTCTGC |
| P62 (Sqstm1) | ATGTGGAACATGGAGGGAAGA | GGAGTTCACCTGTAGATGGGT |
| PPARγ (Pparg) | TCGCTGATGCACTGCCTATG | GAGAGGTCCACAGAGCTGATT |
| C/EBPα (Cebpa) | CAAGAACAGCAACGAGTACCG | GTCACTGGTCAACTCCAGCAC |
| Fabp4 | AAGGTGAAGAGCATCATAACCCT | TCACGCCTTTCATAACACATTCC |
| Scd1 | TTCTTGCGATACACTCTGGTGC | CGGGATTGAATGTTCTTGTCGT |
| Perilipin (Plin1) | CTGTGTGCAATGCCTATGAGA | CTGGAGGGTATTGAAGAGCCG |
| 36b4 | AAGCGCGTCCTGGCATTGTCT | CCGCAGGGGCAGCAGTGGT |

**Supplementary Figure 1:** LC3 content were determined by Western blot in the stromal vascular fraction (SVF) and mature adipocytes of subcutaneous adipose tissue (SAT) from mice fed a high fat diet (HFD) or standard chow diet for 24 weeks.

**Supplementary Figure 2:** Series cluster analysis was introduced to discover the expression trend of DEGs in 3T3-L1 adipocytes with TNFα treatment.

**Supplementary Figure 3:** The SAT and EAT were obtained from mice fed with HFD or standard chow diet for 24 weeks, and the SVF and mature adipocytes were separately isolated from the SAT and EAT. Lysosomal/autophagic genes in adipose tissue (**A**) and in SVF and mature adipocytes (**B**) were determined by qRT-PCR. *: p <0.05, **: p <0.01, ***: p <0.001.

**Supplementary Figure 4:** (**A**) Lysosomal/autophagic genes under TNFα treatment in 3T3-L1 adipocytes and pathway annotated genes in profile 14, 20 and 23 were merged to construct a gene co-expression network. A k-core of a given gene indicates its hub status with connection to “k” other genes in a network (scale shown in the lower right). (**B**-**D**) C57BL/6 mice were fed with HFD or standard chow diet for 24 weeks, then expression levels of Ctsb in SAT (**B**) were determined by western blot. During the differentiation process of 3T3-L1 cells, Ctsb mRNA and protein levels were determined by qRT-PCR (**C**) and Western blot (**D**).

**Supplementary Figure 5:** (**A**) Immunoblot detection of Becn1、Atg12、Atg5、Atg7 and Atg16L1, p62/ SQSTM1 and LC3 in adipocytes treated with TNFα for 24h. (**B**) The SQSTM1 expression in adipocytes with 4 h TNFα treatment in the presence or absence of CQ/BAF was determined by Western blot. (**C**)Adipocytes treated with TNFα for indicated time, and p62/ SQSTM1 expression was determined by qRT-PCR. (**D**-**E**) 3T3L1 cells were infected with lentivirus to overexpress Ctsb and induced differentiated adipocytes. In Ctsb overexpressed 3T3-L1 adipocytes, protein levels of c-caspase3, caspase 3, LC3 and p62/ SQSTM1 were determined by Western blot (**D**). After adipocytes pretreated with 10 μM CA074 for 1 h and stimulated with TNFα for 4 h, Western blot was performed to analyze the status of c-caspase 3 and caspase 3 (**E**). (**F**) mRNA levels of PPARγ, C/EBPα，Fabp4, Scd1 and Perilipin were determined by qRT-PCR. *: p <0.05

**References**

1.Vulevic B, Chen Z, Boyd JT, Davis W, Jr., Walsh ES, Belinsky MG*, et al.* Cloning and characterization of human adenosine 5'-triphosphate-binding cassette, sub-family A, transporter 2 (ABCA2). *Cancer Res* 2001; **61:** 3339-3347.

2.Sun P, Sleat DE, Lecocq M, Hayman AR, Jadot M, Lobel P. Acid phosphatase 5 is responsible for removing the mannose 6-phosphate recognition marker from lysosomal proteins. *Proc Natl Acad Sci U S A* 2008; **105:** 16590-16595.

3.Wu FQ, Fang T, Yu LX, Lv GS, Lv HW, Liang D*, et al.* ADRB2 signaling promotes HCC progression and sorafenib resistance by inhibiting autophagic degradation of HIF1alpha. *J Hepatol* 2016; **65:** 314-324.

4.Wicker G, Prill V, Brooks D, Gibson G, Hopwood J, von Figura K*, et al.* Mucopolysaccharidosis VI (Maroteaux-Lamy syndrome). An intermediate clinical phenotype caused by substitution of valine for glycine at position 137 of arylsulfatase B. *J Biol Chem* 1991; **266:** 21386-21391.

5.Melland-Smith M, Ermini L, Chauvin S, Craig-Barnes H, Tagliaferro A, Todros T*, et al.* Disruption of sphingolipid metabolism augments ceramide-induced autophagy in preeclampsia. *Autophagy* 2015; **11:** 653-669.

6.de Villiers WJ, Smart EJ. Macrophage scavenger receptors and foam cell formation. *J Leukoc Biol* 1999; **66:** 740-746.

7.Aguilera B, Ghauharali-van der Vlugt K, Helmond MT, Out JM, Donker-Koopman WE, Groener JE*, et al.* Transglycosidase activity of chitotriosidase: improved enzymatic assay for the human macrophage chitinase. *J Biol Chem* 2003; **278:** 40911-40916.

8.Mole SE. The genetic spectrum of human neuronal ceroid-lipofuscinoses. *Brain Pathol* 2004; **14:** 70-76.

9.Yan CH, Li Y, Tian XX, Zhu N, Song HX, Zhang J*, et al.* CREG1 ameliorates myocardial fibrosis associated with autophagy activation and Rab7 expression. *Biochim Biophys Acta* 2015; **1852:** 353-364.

10.Ha SD, Ham B, Mogridge J, Saftig P, Lin S, Kim SO. Cathepsin B-mediated autophagy flux facilitates the anthrax toxin receptor 2-mediated delivery of anthrax lethal factor into the cytoplasm. *J Biol Chem* 2010; **285:** 2120-2129.

11.Goussetis DJ, Gounaris E, Wu EJ, Vakana E, Sharma B, Bogyo M*, et al.* Autophagic degradation of the BCR-ABL oncoprotein and generation of antileukemic responses by arsenic trioxide. *Blood* 2012; **120:** 3555-3562.

12.Huang CC, Chen KL, Cheung CH, Chang JY. Autophagy induced by cathepsin S inhibition induces early ROS production, oxidative DNA damage, and cell death via xanthine oxidase. *Free Radic Biol Med* 2013; **65:** 1473-1486.

13.HGNC. EPDR1 ependymin related 1 [ Homo sapiens (human) ]. 2017 [cited]Available from: https://[www.ncbi.nlm.nih.gov/gene/54749](http://www.ncbi.nlm.nih.gov/gene/54749)

14.Milan G, Romanello V, Pescatore F, Armani A, Paik JH, Frasson L*, et al.* Regulation of autophagy and the ubiquitin-proteasome system by the FoxO transcriptional network during muscle atrophy. 2015; **6:** 6670.

15.Ganley IG. Autophagosome maturation and lysosomal fusion. *Essays Biochem* 2013; **55:** 65-78.

16.Da Ros M, Lehtiniemi T, Olotu O, Fischer D, Zhang FP, Vihinen H*, et al.* FYCO1 and autophagy control the integrity of the haploid male germ cell-specific RNP granules. *Autophagy* 2017; **13:** 302-321.

17.Shea L, Raben N. Autophagy in skeletal muscle: implications for Pompe disease. *Int J Clin Pharmacol Ther* 2009; **47 Suppl 1:** S42-47.

18.Nascimbeni AC, Fanin M, Masiero E, Angelini C, Sandri M. The role of autophagy in the pathogenesis of glycogen storage disease type II (GSDII). *Cell Death Differ* 2012; **19:** 1698-1708.

19.Xu YH, Barnes S, Sun Y, Grabowski GA. Multi-system disorders of glycosphingolipid and ganglioside metabolism. *J Lipid Res* 2010; **51:** 1643-1675.

20.Xu M, Motabar O, Ferrer M, Marugan JJ, Zheng W, Ottinger EA. Disease models for the development of therapies for lysosomal storage diseases. *Ann N Y Acad Sci* 2016; **1371:** 15-29.

21.Fedele AO. Sanfilippo syndrome: causes, consequences, and treatments. *Appl Clin Genet* 2015; **8:** 269-281.

22.Holler CJ, Taylor G, McEachin ZT, Deng Q, Watkins WJ, Hudson K*, et al.* Trehalose upregulates progranulin expression in human and mouse models of GRN haploinsufficiency: a novel therapeutic lead to treat frontotemporal dementia. 2016; **11:** 46.

23.Lepperdinger G, Strobl B, Kreil G. HYAL2, a human gene expressed in many cells, encodes a lysosomal hyaluronidase with a novel type of specificity. *J Biol Chem* 1998; **273:** 22466-22470.

24.Lopez-Herrera G, Tampella G, Pan-Hammarstrom Q, Herholz P, Trujillo-Vargas CM, Phadwal K*, et al.* Deleterious mutations in LRBA are associated with a syndrome of immune deficiency and autoimmunity. *Am J Hum Genet* 2012; **90:** 986-1001.

25.Gan-Or Z, Dion PA, Rouleau GA. Genetic perspective on the role of the autophagy-lysosome pathway in Parkinson disease. *Autophagy* 2015; **11:** 1443-1457.

26.Bouche V, Espinosa AP, Leone L, Sardiello M, Ballabio A, Botas J. Drosophila Mitf regulates the V-ATPase and the lysosomal-autophagic pathway. *Autophagy* 2016; **12:** 484-498.

27.Perera RM, Stoykova S, Nicolay BN, Ross KN, Fitamant J, Boukhali M*, et al.* Transcriptional control of autophagy-lysosome function drives pancreatic cancer metabolism. *Nature* 2015; **524:** 361-365.

28.Heraud C, Griffiths A, Pandruvada SN, Kilimann MW, Pata M, Vacher J. Severe neurodegeneration with impaired autophagy mechanism triggered by ostm1 deficiency. *J Biol Chem* 2014; **289:** 13912-13925.

29.Ivankovic D, Chau KY, Schapira AH, Gegg ME. Mitochondrial and lysosomal biogenesis are activated following PINK1/parkin-mediated mitophagy. *J Neurochem* 2016; **136:** 388-402.

30.Roberts RF, Fon EA. Presenting mitochondrial antigens: PINK1, Parkin and MDVs steal the show. *Cell Res* 2016; **26:** 1180-1181.

31.Sun Y, Liou B, Ran H, Skelton MR, Williams MT, Vorhees CV*, et al.* Neuronopathic Gaucher disease in the mouse: viable combined selective saposin C deficiency and mutant glucocerebrosidase (V394L) mice with glucosylsphingosine and glucosylceramide accumulation and progressive neurological deficits. *Hum Mol Genet* 2010; **19:** 1088-1097.

32.Takematsu H, Diaz S, Stoddart A, Zhang Y, Varki A. Lysosomal and cytosolic sialic acid 9-O-acetylesterase activities can Be encoded by one gene via differential usage of a signal peptide-encoding exon at the N terminus. *J Biol Chem* 1999; **274:** 25623-25631.

33.Aizawa S, Fujiwara Y, Contu VR, Hase K, Takahashi M, Kikuchi H*, et al.* Lysosomal putative RNA transporter SIDT2 mediates direct uptake of RNA by lysosomes. *Autophagy* 2016; **12:** 565-578.

34.Bissa B, Beedle AM, Govindarajan R. Lysosomal solute carrier transporters gain momentum in research. *Clin Pharmacol Ther* 2016; **100:** 431-436.

35.Sagne C, Agulhon C, Ravassard P, Darmon M, Hamon M, El Mestikawy S*, et al.* Identification and characterization of a lysosomal transporter for small neutral amino acids. *Proc Natl Acad Sci U S A* 2001; **98:** 7206-7211.

36.Bond CS, Clements PR, Ashby SJ, Collyer CA, Harrop SJ, Hopwood JJ*, et al.* Structure of a human lysosomal sulfatase. *Structure* 1997; **5:** 277-289.

37.Mullock BM, Smith CW, Ihrke G, Bright NA, Lindsay M, Parkinson EJ*, et al.* Syntaxin 7 is localized to late endosome compartments, associates with Vamp 8, and Is required for late endosome-lysosome fusion. *Mol Biol Cell* 2000; **11:** 3137-3153.

38.Martina JA, Diab HI, Lishu L, Jeong AL, Patange S, Raben N*, et al.* The nutrient-responsive transcription factor TFE3 promotes autophagy, lysosomal biogenesis, and clearance of cellular debris. *Sci Signal* 2014; **7:** ra9.

39.Steingrimsson E, Tessarollo L, Pathak B, Hou L, Arnheiter H, Copeland NG*, et al.* Mitf and Tfe3, two members of the Mitf-Tfe family of bHLH-Zip transcription factors, have important but functionally redundant roles in osteoclast development. *Proc Natl Acad Sci U S A* 2002; **99:** 4477-4482.

40.Rehli M, Sulzbacher S, Pape S, Ravasi T, Wells CA, Heinz S*, et al.* Transcription factor Tfec contributes to the IL-4-inducible expression of a small group of genes in mouse macrophages including the granulocyte colony-stimulating factor receptor. *J Immunol* 2005; **174:** 7111-7122.

41.He P, Peng Z, Luo Y, Wang L, Yu P, Deng W*, et al.* High-throughput functional screening for autophagy-related genes and identification of TM9SF1 as an autophagosome-inducing gene. *Autophagy* 2009; **5:** 52-60.

42.Liang C, Feng P, Ku B, Dotan I, Canaani D, Oh BH*, et al.* Autophagic and tumour suppressor activity of a novel Beclin1-binding protein UVRAG. *Nat Cell Biol* 2006; **8:** 688-699.
