# Supplementary figures and images for: Obesity-associated inflammation triggers an autophagy–lysosomal response in adipocytes and causes degradation of perilipin 1
Source: Cell Death Dis. 2019 Feb 11;10(2):121. doi: 10.1038/s41419-019-1393-8 (PMC6370809; doi:10.1038/s41419-019-1393-8)

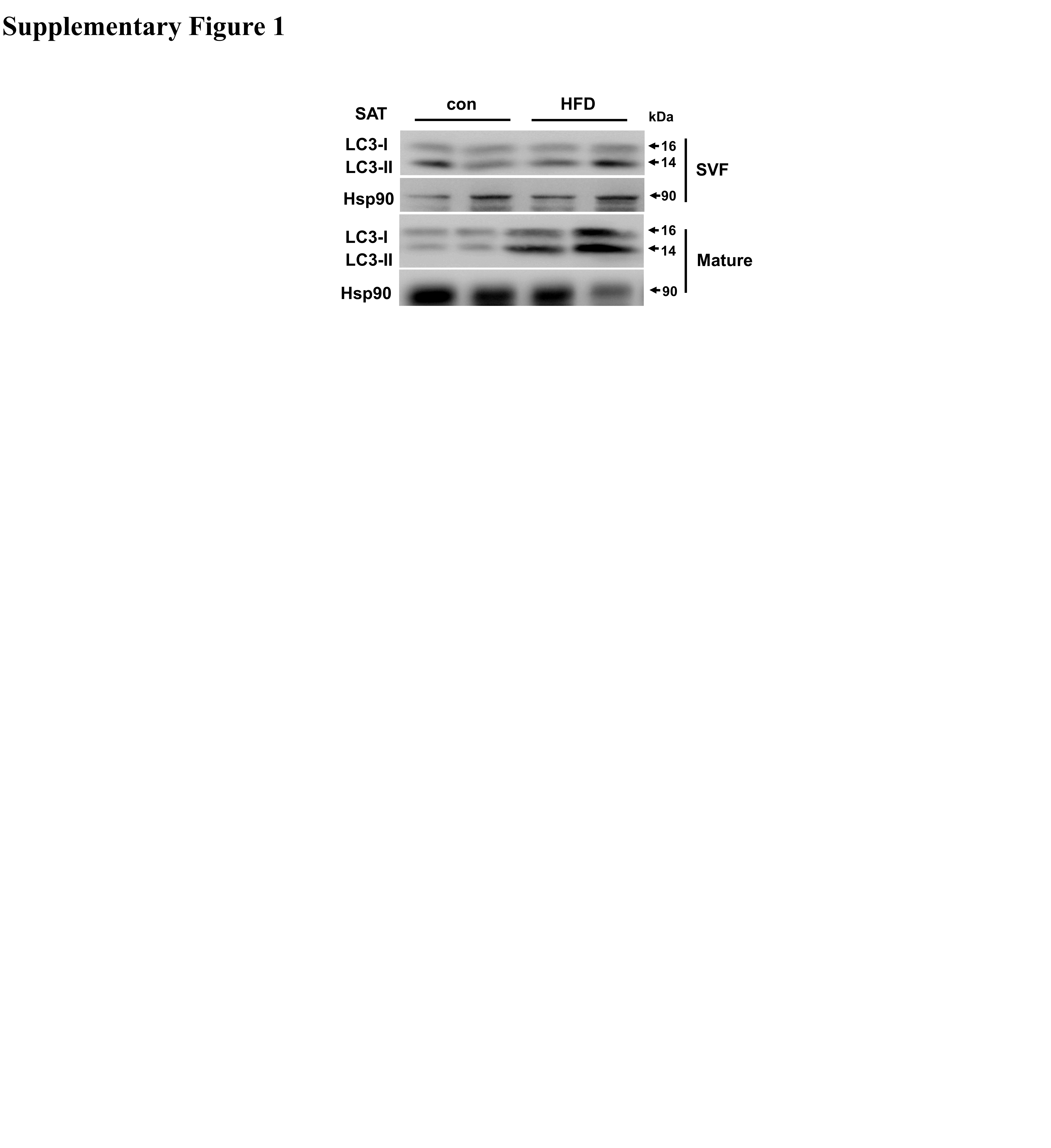

Supplement: Supplementary file 2 — Figure S1 [file 41419_2019_1393_MOESM2_ESM.tif]

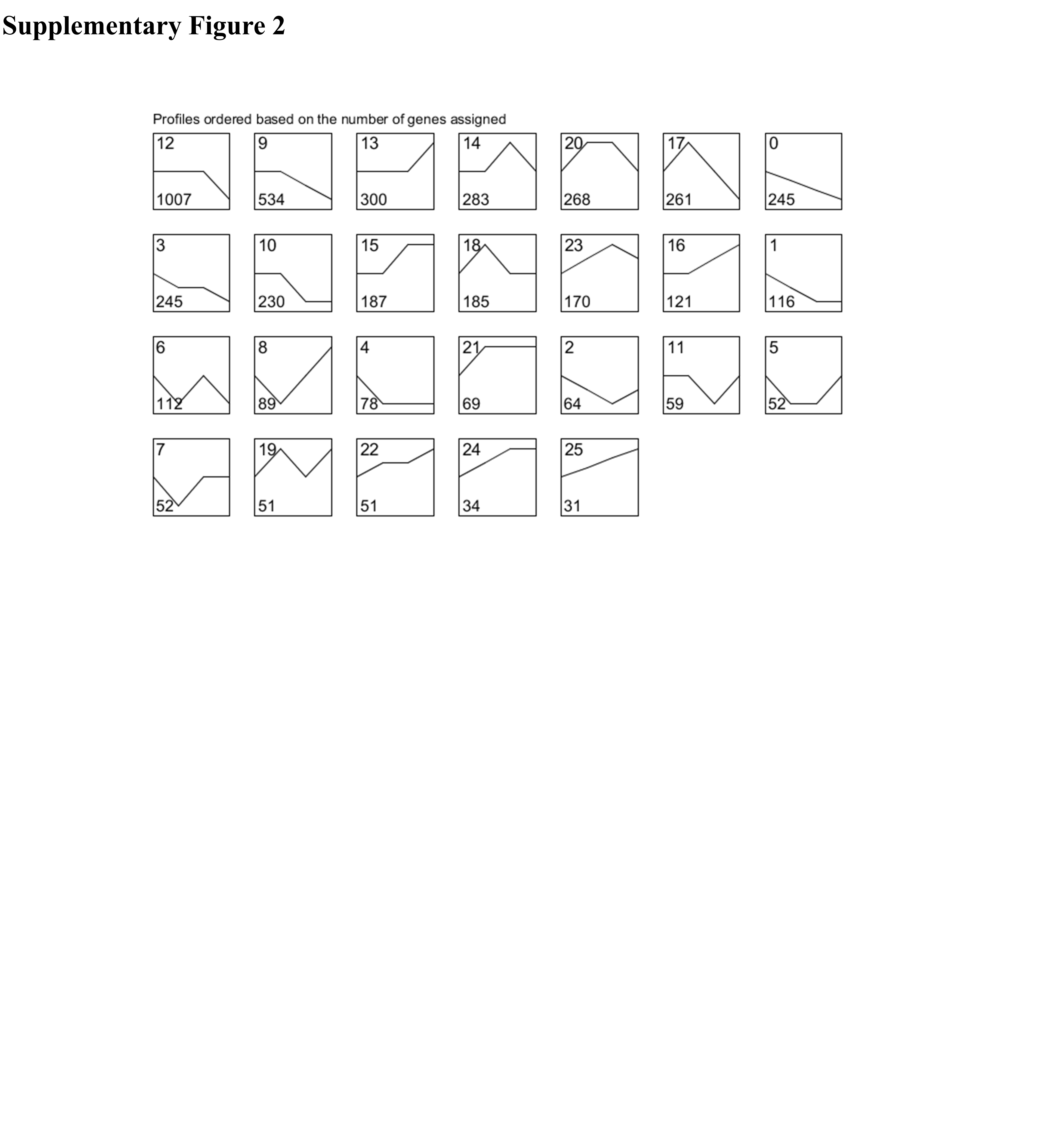

Supplement: Supplementary file 3 — Figure S2 [file 41419_2019_1393_MOESM3_ESM.tif]

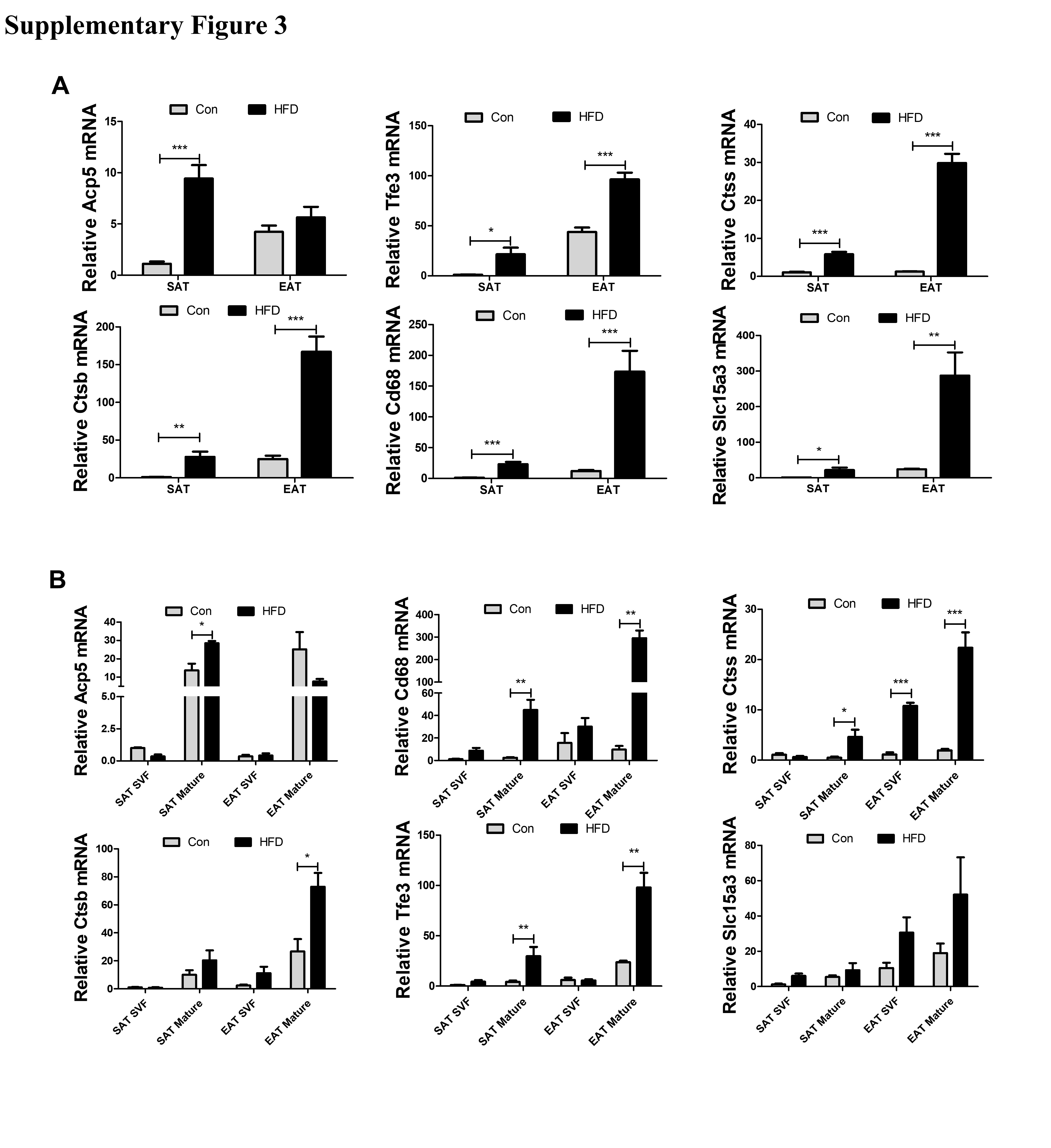

Supplement: Supplementary file 4 — Figure S3 [file 41419_2019_1393_MOESM4_ESM.tif]

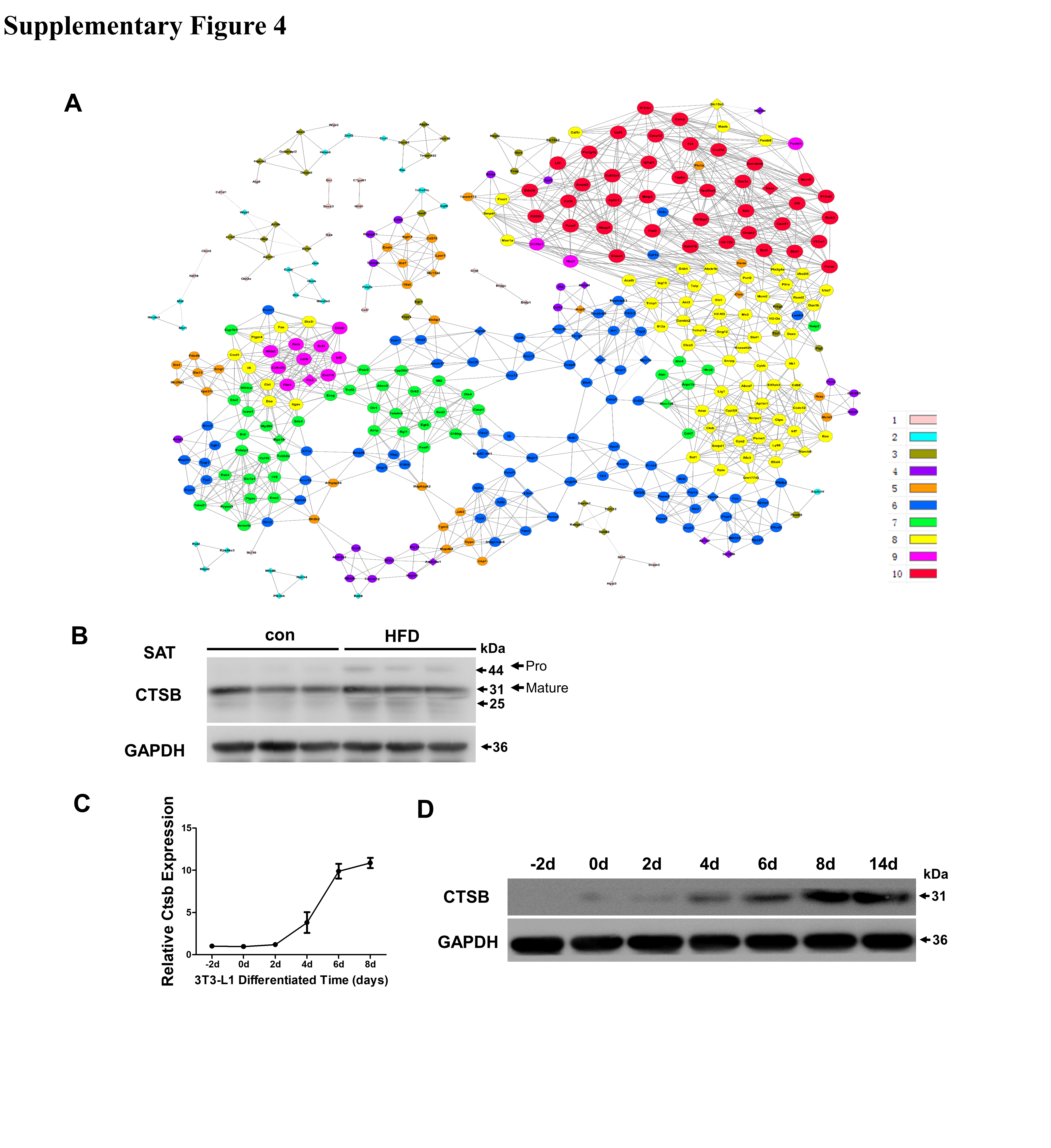

Supplement: Supplementary file 5 — Figure S4 [file 41419_2019_1393_MOESM5_ESM.tif]

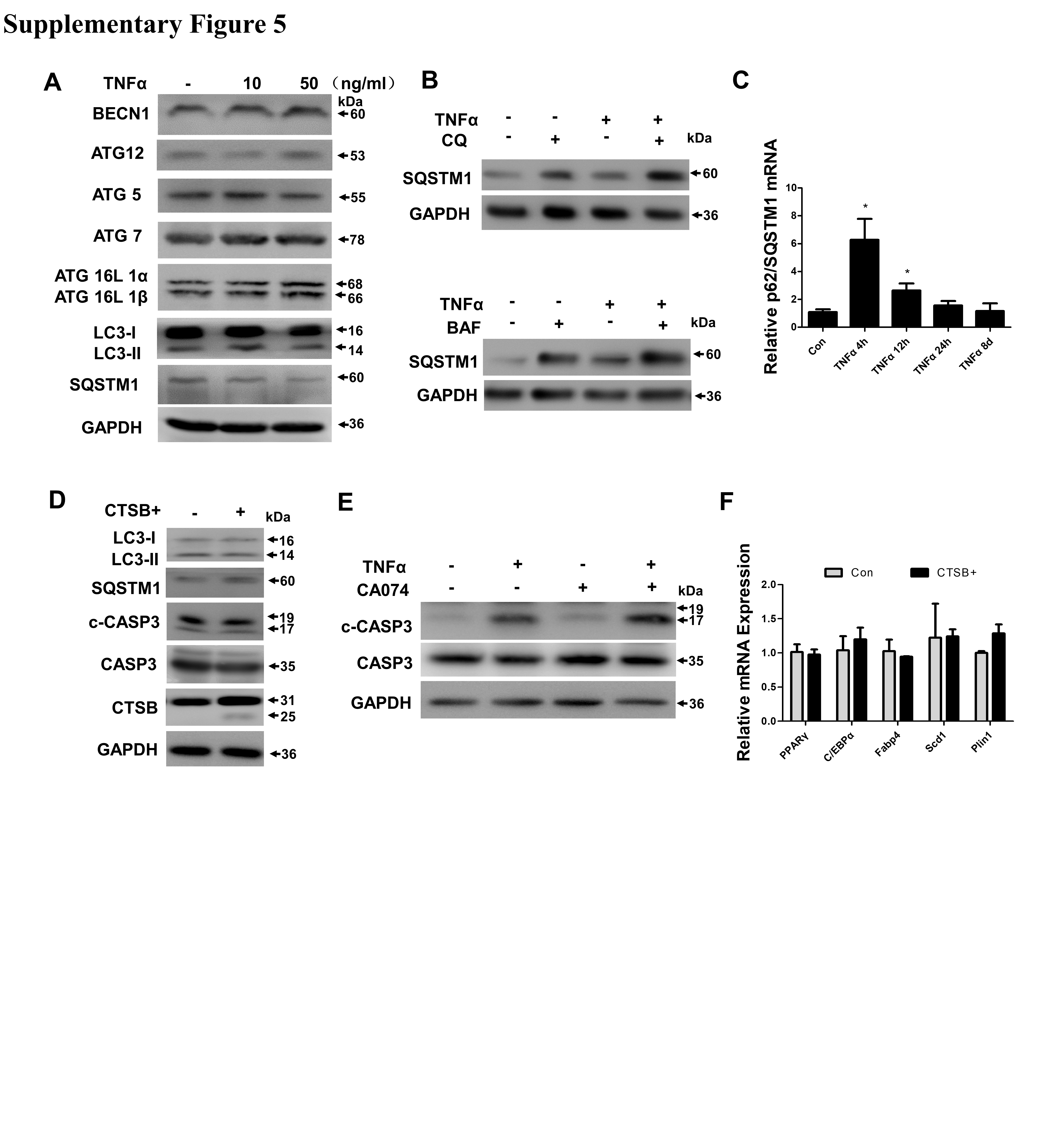

Supplement: Supplementary file 6 — Figure S5 [file 41419_2019_1393_MOESM6_ESM.tif]
